# Supplementary figures and images for: Relative contributions of the correlates of stunting in explaining the mean length-for-age z-score difference between 24-month-old stunted and non-stunted children living in a slum of Dhaka, Bangladesh: results from a decomposition analysis
Source: BMJ Open. 2019 Jul 30;9(7):e025439. doi: 10.1136/bmjopen-2018-025439 (PMC6678062; doi:10.1136/bmjopen-2018-025439)

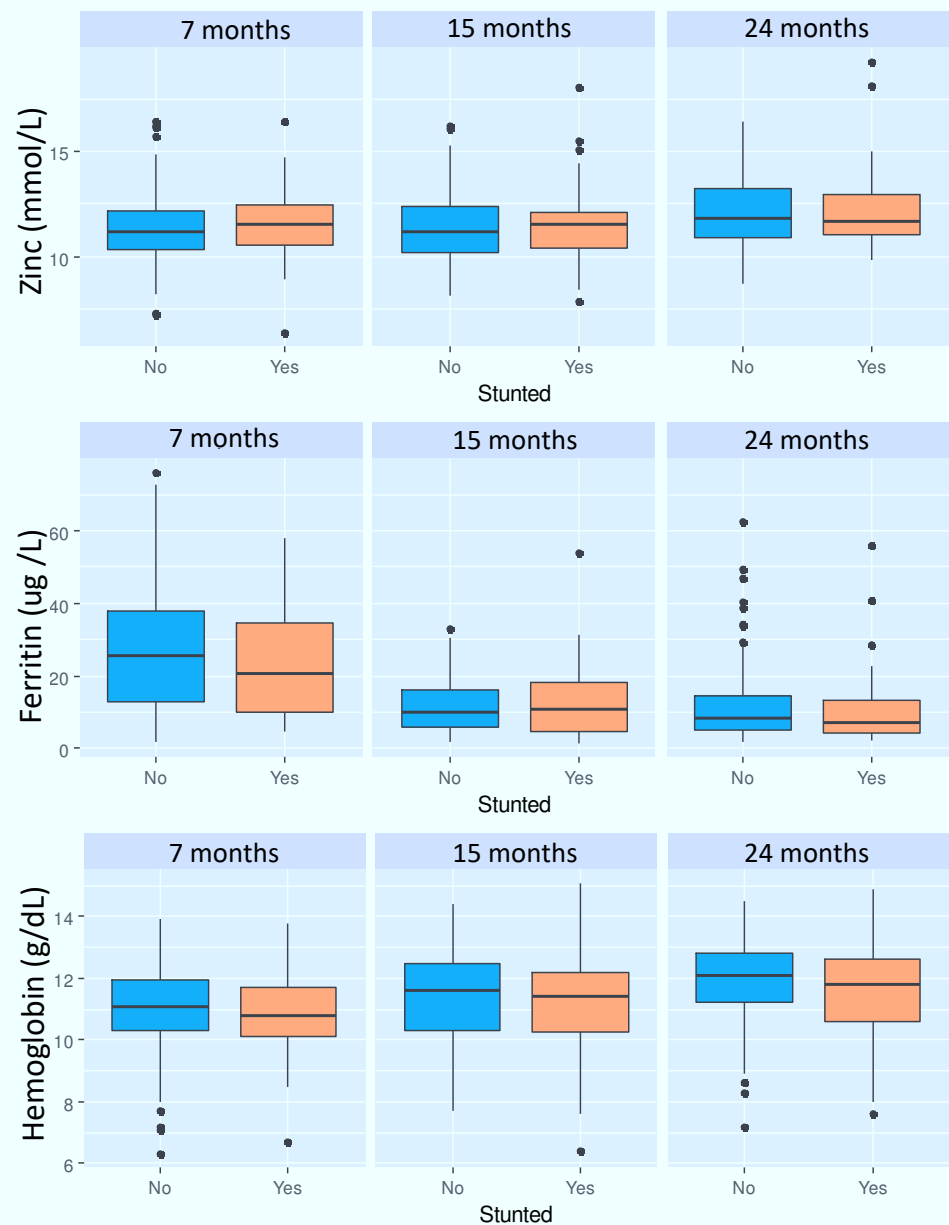

Supplement: Supplementary data [file bmjopen-2018-025439supp001.pdf]

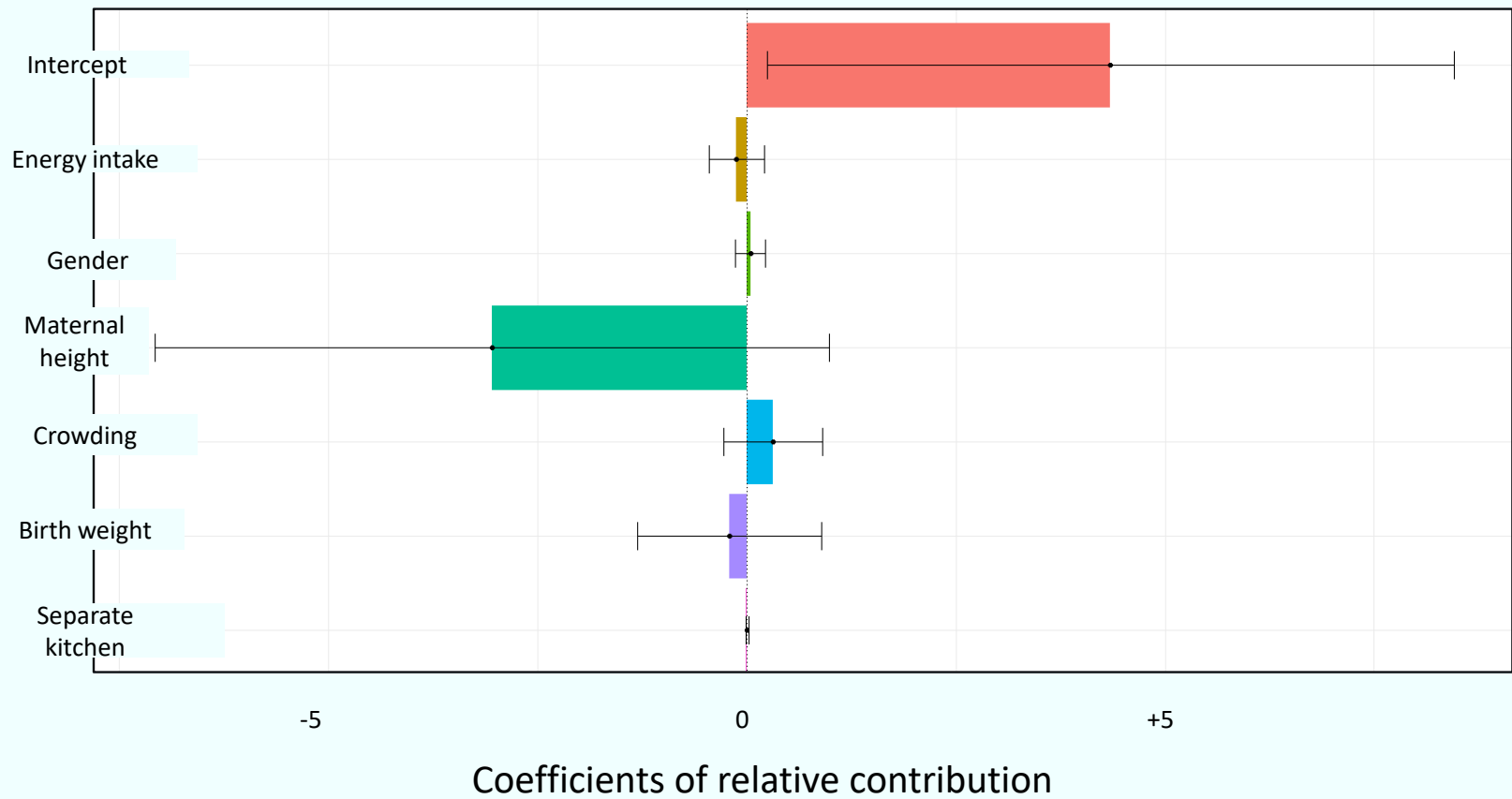

Supplement: Supplementary data [file bmjopen-2018-025439supp002.pdf]
