# Supplementary material for: Relative contributions of the correlates of stunting in explaining the mean length-for-age z-score difference between 24-month-old stunted and non-stunted children living in a slum of Dhaka, Bangladesh: results from a decomposition analysis
Source: BMJ Open. 2019 Jul 30;9(7):e025439. doi: 10.1136/bmjopen-2018-025439 (PMC6678062; doi:10.1136/bmjopen-2018-025439)
Supplement: Author's manuscript [file bmjopen-2018-025439.draft_revisions.pdf]

BMJ Open is committed to open peer review. As part of this commitment we make the peer review history of every article we publish publicly available.

When an article is published we post the peer reviewers' comments and the authors' responses online. We also post the versions of the paper that were used during peer review. These are the versions that the peer review comments apply to.

The versions of the paper that follow are the versions that were submitted during the peer review process. They are not the versions of record or the final published versions. They should not be cited or distributed as the published version of this manuscript.

BMJ Open is an open access journal and the full, final, typeset and author-corrected version of record of the manuscript is available on our site with no access controls, subscription charges or pay-per-view fees (http://bmjopen.bmj.com).

If you have any questions on BMJ Open's open peer review process please email info.bmjopen@bmj.com

BMJ Open

To what extent do the determinants of stunting contribute to mean length-for-age Z score difference between stunted and non-stunted children at 24 months of age? Results from a decomposition analysis using data from a birth cohort study conducted in Dhaka, Bangladesh.

Journal:	*BMJ Open*
Manuscript ID	bmjopen-2018-025439
Article Type:	Research
Date Submitted by the Author:	17-Jul-2018

Complete List of Authors:	Das, Subhasish; International Centre for Diarrhoeal Disease Research, Bangladesh (icddr,b), Nutrition and Clinical Services Division; Employer<br>Alam, Md Ashraful; International Centre for Diarrhoeal Disease Research Bangladesh, Nutrition and Clinical Services Division<br>Mahfuz, Mustafa; International Centre for Diarrhoeal Disease Research, Nutrition and Clinical Services Division<br>Arifeen, Shams E.; ICDDR B, Maternal and Child Health Division (MCHD)<br>Ahmed, Tahmeed; International Centre for Diarrhoeal Disease Research, Bangladesh (icddr,b), Nutrition and Clinical Services Division (NCSD)

Keywords:	Length-for-age Z (LAZ) score, Relative contribution, Decomposition analysis, Maternal height, Stunting

SCHOLARONE™
Manuscripts

For peer review only

TITLE: To what extent do the determinants of stunting contribute to mean length-for-age Z score difference between stunted and non-stunted children at 24 months of age? Results from a decomposition analysis using data from a birth cohort study conducted in Dhaka, Bangladesh.

Corresponding author:

Subhasish Das, Nutrition and Clinical Services Division, International Centre for Diarrhoeal Disease Research Bangladesh (icddr,b), Dhaka 1212, Bangladesh. Email: subhasish.das@icddrb.org. Telephone number: +8801617099766

Co-authors:

Md Ashraful Alam, Nutrition and Clinical Services Division, icddr,b, Dhaka, Bangladesh

Mustafa Mahfuz, Nutrition and Clinical Services Division, icddr,b, Dhaka, Bangladesh

Shams El Arifeen, Maternal and Child Health Division, icddr,b, Dhaka, Bangladesh

Tahmeed Ahmed, Nutrition and Clinical Services Division, icddr,b, Dhaka, Bangladesh

Authors' email addresses: subhasish.das@icddrb.org, mashraful@icddrb.org, mustafa@icdddrb.org, shams@icddrb.org, tahmeed@icddrb.org

Body text word count: 2636

Keywords: Stunting, Length-for-age Z (LAZ) score, Relative contribution, Decomposition analysis, Maternal height.

ABSTRACT

Objective: Identifying the specific contribution of the determinants of stunting can help the policy makers to design specific interventions. Using Mal-ED birth cohort data we sought to measure the

For peer review only

relative contribution of the determinants of stunting on mean length-for-age Z (LAZ) score difference of stunted and non-stunted children at their 24 months of age.

Setting: Dhaka, Bangladesh

Participants: 211 slum dwelling children enrolled within 17 days of their birth.

Variables and method: The explanatory variables were identified from following groups: maternal characteristics, birth characteristics, macronutrient intake, socio-economic status, morbidity and serum micronutrient level. At step 1, determinants of stunting were identified longitudinally using Generalized Estimating Equations (GEE) model. Then, relative contribution of identified variables on mean LAZ score difference (outcome variable) between stunted and non-stunted children at their 24 month was measured using Blinder-Oaxaca decomposition analysis.

Results: At 24 month the prevalence of stunting was 47.9% and mean LAZ score difference between stunted (mean LAZ -2.81) and non-stunted children (mean LAZ -1.33) was 1.48. The GEE multivariable model identified maternal height (OR 0.87, 95% CI: 0.89-0.86), birth weight (OR 0.11, 95% CI: 0.15-0.09), people per room (OR 1.48, 95% CI: 1.62-1.35), gender (female, OR 0.47, 95% CI: 0.58-0.38), and having separate room for kitchen (OR 0.21, 95% CI: 0.38-0.12) as the determinants of stunting. Variable by variable decomposition of the LAZ score difference between stunted and non-stunted children at 24 months identified maternal height (endowment: 0.1 ; 95% CI: 0.18,0.02), birth weight (endowment: 0.08; 95% CI: 0.17,-0.01) and people per room (endowment: 0.06 ; 95% CI: 0.12,0.00) to be the top most contributors.

Conclusions: The relatively strong contribution of maternal height and birth weight on childhood stunting affirms the intergenerational role and importance of nutrition programs for adolescent girls and women of reproductive age.

For peer review only

For peer review only - http://bmjopen.bmj.com/site/about/guidelines.xhtml

Strengths and limitations of this study

- This study uses the Blinder-Oaxaca decomposition analysis to measure the relative contribution of different determinants of stunting on LAZ score difference between stunted and non-stunted children.
- The threefold Blinder-Oaxaca decomposition technique is used to decompose the main outcome into three components: endowments, coefficients and the interaction of the two.
- The explanatory variables that were used for decomposition analysis were identified after analyzing the longitudinal data collected from the same cohort of children.
- Among the limitations, we excluded the anthropometry data collected before 9 months of age to ensure the temporality of longitudinal analysis as dietary intake data collection started at 9 months.
- Serum micronutrient data was collected at 7, 15 and 24 months.

INTRODUCTION

Stunting (length-for-age more than two standard deviations below the WHO child growth standards median) is an indicator of chronic undernutrition and is one of the best measures of child health inequality [1]. It is the most prevalent form of malnutrition which is responsible for higher mortality and cognitive impairment of affected children [1-4]. Worldwide 155 million under 5 children are stunted and in Asia the number is a staggering 87 million [5]. Variation in rates of stunting exists not only across continents but also within local geographies [6]. The prevalence of stunting in Bangladeshi slum areas is much higher than the national level average (50 vs. 36 %, respectively) [7 8].

Children from lowest wealth quintiles who have a normal anthropometric status at birth may experience an intense period of growth retardation which may continue up to the third year of life or longer [9].

For peer review only - http://bmjopen.bmj.com/site/about/guidelines.xhtml

During the subsequent life-cycle stages this growth retardation continues to exert negative effects such as socio-emotional impairment in pre-school age, poor school attainment in early childhood, and higher risk of chronic disease in adulthood [10]. Multiple studies have reported recurrent infection, insufficient food intake and poor socio-economic condition to be the factors responsible for poor linear growth; but none of these studies reported the relative contribution of the determinants on length-for-age Z (LAZ) score difference of stunted and non-stunted children [5-7].

Keeping the context in mind, we aimed to examine the relative contribution of the determinants of stunting on mean LAZ score difference between stunted and non-stunted children at 24 months. In high burden countries the benefit-cost ratio for investments to reduce stunting ranges from 3.8 to 47.9 and in Bangladesh the ratio is 18.4[11 12]. Being a resource constraints country, identification of top contributors of stunting will allow policy-makers to invest on specific segments of the life course of a population and thus make better use of resources.

METHODS

Study participants

Data used for this analysis was collected from the MAL-ED (Etiology, Risk Factors, and Interactions of Enteric Infections and Malnutrition and the Consequences for Child Health) Bangladesh site. The MAL-ED birth cohort study was conducted at 8 sites in 3 continents. In Bangladesh the MAL-ED study site was Bauniabadh slum, Mirpur, Dhaka which was described elsewhere[13]. Following a well-defined protocol, field workers visited households and enrolled 265 healthy newborn children within 17 days of their birth between February 2010 and February 2012. Participants were followed till 24 months of age. At 24 months, 211 participants remained which is 80% of total recruitment. The study was approved by the Institutional Review Board of icddr,b (protocol no. PR-2008-020).

For peer review only - http://bmjopen.bmj.com/site/about/guidelines.xhtml

For peer review only

Data collection

Dependent variable

Following standard anthropometric methodology trained field workers measured recumbent length of the children to the nearest 1 mm using Seca Infantometer (model no.417, Hamburg, Germany) [14]. The LAZ score of each child was determined using the WHO 2006 Child Growth Standards [15]. A child with LAZ < –2 is classified as stunted [15].

Independent variables

The explanatory variables identified for the analysis were drawn from the following groups: birth characteristics, maternal characteristics, nutrient intake, socio-economic status, morbidity and serum micronutrient. The birth characteristics data (enrollment weight and length) and maternal height was measured within 72 h after recruitment. Children were weighed with minimum clothing using a digital scale with 10 g precision (Seca, model no.345, Hamburg, Germany). Enrollment weight was used as the surrogate measure for birthweight. Standard wooden height measuring boards were used for measuring maternal height. For assessing feeding habit and energy intake of the children, 24 hours food frequency data was collected monthly from 9th month onwards using a 24-hour multiple-pass dietary recall approach[16]. The 24-hr recall was conducted on non-consecutive days and out of every 4 recalls, 1 interview was conducted on a weekend. The dietary intake data were converted to energy using a locally adapted food composition table. Infant and Young Child Feeding Practice was evaluated using Minimum Acceptable Diet (MAD) indicator as it meets standards for both minimum dietary diversity and minimum meal frequency[17]. Socio-economic data was collected at 6, 12, 18 and 24 months. WAMI index (ranging from 0 to 1) -a socio-economic status index which includes access to improved water and sanitation, eight selected assets, maternal education, and household income was used as a

For peer review only

For peer review only - http://bmjopen.bmj.com/site/about/guidelines.xhtml

representative of socio-economic status variables[18].To measure the household level crowding a continuous variable named "people per room" was generated from number of rooms in the household that were used for sleeping and total number of people usually sleep in the household. Blood samples for measuring serum zinc, ferritin and hemoglobin status were collected at 7, 15 and 24 months of age. For hemoglobin, one drop of capillary blood was collected and measured with the Hemocue device (Hb 201, Ängelholm, Sweden). Plasma zinc and Ferritin levels were measured using Atomic Absorption Spectometry (AAS) and Chemiluminescence Immunoassay (CLIA), respectively. Anemia is defined as a hemoglobin concentration <110 g/L [19]. Plasma ferritin and zinc concentration values were adjustment for the effect of subclinical inflammation using both C-reactive protein (CRP) and α-1-acid glycoprotein (AGP) [20 21].

Data on morbidity and infection status was collected twice a week. Diarrhea is defined as having ≥3 loose stools in a 24-hour period, or at least 1 loose stool with blood reported by the mother [22]. A diarrheal episode is defined as being separated from another episode by at least 2 or more diarrhea-free days [22-24]. Acute lower respiratory infection (ALRI) is identified based on the presence of cough and/or shortness of breath plus high respiratory rate [24]. The average of the 2 respiratory rates was used, and was considered high based on the age of the child (<2 months of age: ≥60 breaths/minute; 2 to <12 months of age: ≥50 breaths/minute; ≥12 months of age: ≥40 breaths/minute) [25]. ALRI episodes are separated by at least 15 ALRI-free days [24]. Fever is defined as an axillary temperature >37.5°C [26].

Statistics

Means and standard deviation are presented for normally distributed continuous variables; frequency and percentage for categorical variables. The association between each explanatory variable and outcome variable (stunting: no vs. yes) was examined longitudinally using Generalized Estimation Equations (GEE) model [27]. The GEE models was constructed using unstructured covariance matrix with

For peer review only

For peer review only - http://bmjopen.bmj.com/site/about/guidelines.xhtml

robust variance estimates. The explanatory variables showing statistically significant association ($p < 0.05$) with the outcome variable were selected for multivariable analysis. Variables with a low frequency and those with multicollinearity (VIF > 5 were excluded from the analysis. Serum micronutrient data was collected at 7, 15 and 24 month. So, we carry forwarded 7 months data up to 14 month, 15 months data up to 23 months. Dietary intake data collection started at 9 months. So, to ensure the availability of maximum variables at every time point and temporality of the relation between outcome and explanatory variables, the multivariable analysis was done using the data collected from 9 to 24 month. Variables showing significant association with stunting in the multivariable GEE model were selected for decomposition analysis. Blinder-Oaxaca decomposition analysis was done to examine the relative contribution of the identified variables on mean length-for-age Z score difference between stunted and non-stunted children at 24 months[28]. During decomposition analysis bootstrapped standard errors were calculated based on 1,000 replicates. R version 3.4.2 was used for data analysis and GEE and Oaxaca packages were employed [29 30].

Patient and Public Involvement (PPI) statement

No patient or public were involved with the development of research question, designing the study, recruitment of participants, interpretation of the results, and will be involved during disseminating the findings of the paper.

RESULTS

At month 1 the cohort exhibited a 19% prevalence of stunting. The prevalence showed a similar trend till month 6 (18.4 %). After that, this trend line went up to 33.6%, 46.8% and 47.9% at 12, 18 and 24th months, respectively.

For peer review only - http://bmjopen.bmj.com/site/about/guidelines.xhtml

The socio-demographic characteristics of the participating households are described in table 1. Till 24 months, the study was able to follow a cohort of 211 children. At 24 months, the overall male to female ratio was 1.04, which was 1.3 in stunted cohort. Only 10.9% of children were exclusively breastfed (received only breast milk for the entire first 6 months). This prevalence did not change over the stunting status. Most of the households were headed by a male family member. 84.4% mothers had an age between 18-30 years. 39.8% mothers were younger than 18 years during their first pregnancy, the percentage of which was less in stunted cohort than in non-stunted cohort. Overall 19.4% mothers were illiterate, nearly half had an education less than 5 years and 36.5% had more than 5 years of education. 8.1% households had separate spaces for kitchen. Nearly half of the households had a monthly income less than 100 USD the percentage of which was higher in stunted group than non-stunted group. Only 64% caregivers treated water to make it safe. On average, most of the children had >3 persons sharing the living room, the rate of which was more in stunted households than non-stunted households. Mean (SD) birth weight of the cohort was 2.82 (0.42) kg which was lower in stunted than non-stunted children. The mothers' of the stunted children also had a lower height than the non-stunted children.

Morbidity data displayed in table 1 show that per year stunted children suffered from more than 4 diarrheal episodes which was higher than the overall rate of 3.63 episodes per year. Similar trends were seen for ALRI and fever episodes (0.54 vs. 0.47 and 8.78 vs. 8.06, respectively).

Figure 1 represents the pattern of macronutrient intake from 9 to 24 month. Over the period ,the trend of nutrient intake did not differ much between stunted and non-stunted groups. For both the groups, carbohydrate was the main source of nutrition.

Figure 2 presents the comparative picture of stunting prevalence among the groups of children who did not take minimum acceptable diet (MAD) and those who took it. At 7 months the non-MAD group had a 21% prevalence of stunting which went up to 54% at 24 months. The MAD group followed a similar

For peer review only - http://bmjopen.bmj.com/site/about/guidelines.xhtml

pattern in prevalence of stunting (19% at 7 months, 46% at 24 months), but every month the non-MAD group had a higher prevalence of stunting than the MAD group.

Box plots reported in supplementary figure 1 shows the comparative picture of serum zinc, ferritin and hemoglobin level of stunted and non-stunted children at 7, 15, and 24 months. Over the months, only serum zinc level showed statistically significant (p value <0.05) difference between stunted and non-stunted children.

For peer review only

Table 2 shows the results of generalized estimating equation modeling. A priori conceptual framework was developed for identifying the variables of interest for analysis. Based on the conceptual framework, bivariate analysis was carried out longitudinally by taking into account all the variables, one at a time. However, table 2 shows only the variables having a statistically significant association (p value <0.05) with stunting. From nutrient intake group the amount of energy intake was selected because separate food groups (carbohydrate, total protein, animal protein and fat) showed a very high correlation with each other. Instead of exclusive breast feeding (ebf) status (yes vs. no), exclusive breast feeding days (ebf days) was included for the final model as it counts the specific number of days. The multivariable model showed significant (p value < 0.05) association of energy intake, female gender, maternal height, having a separate room for kitchen, people per room and birth weight with stunting.

Table 3 describes the result of decomposition analysis and supplementary figure 2 graphically displays the coefficient values and 95% confidence intervals of endowment, coefficient and interception for overall model and also for specific variables. At 24 months the mean LAZ for non-stunted group was -1.33 and in stunted group it was -2.81, yielding a LAZ gap of 1.48. The results of the threefold decomposition suggest that, of the 1.48 difference, approximately 0.26 can be attributed to group differences in endowments (i.e., maternal height, birth weight, people per room), 1.34 to differences in coefficients, and the remaining -0.12 is accounted for by the interaction of the two. The 'endowment'

For peer review only - http://bmjopen.bmj.com/site/about/guidelines.xhtml

part of the result reflects that if stunted children had the same characteristics as non-stunted, their mean LAZ would be 0.26 more than the reported one which is -2.81. This also indicates that differences in the magnitude or extent of explanatory variables account for about 16% of the LAZ gap among stunted and non-stunted children. If the non-stunted cohort's coefficients (effect) were applied to the stunted cohort's characteristics the changes in stunted cohort's LAZ would be 1.34. Next, the variable by variable decomposition of this LAZ gap identifies maternal height, birth weight and people per room as the top most contributors.

DISCUSSION

At 24 months the cohort exhibited 47.9% prevalence of stunting. The figure is similar to Bangladesh Urban Health Survey which reported a 50% prevalence of stunting in under 5 children living in slum areas of Dhaka, Bangladesh [8].

Our analysis found maternal height to be inversely associated with stunting and exerting the highest relative contribution in LAZ difference between stunted and non-stunted children at 24 month. Comparable findings were reported from studies conducted in similar settings. Results from 109 Demographic and Health Surveys conducted in 54 LMICs between 1991 and 2008 found maternal stature to be inversely associated with stunting during infancy and childhood [31]. This could be explained by the biological plausibility that shorter mothers might have a poorer health and nutrition status and failed to deliver adequate nutrition to the fetus during pregnancy [32]. This finding signifies the intergenerational effect of malnutrition.

The negative association between birth weight and stunting identifies it as another important determinant. Studies conducted in Burundi, Nepal, Tanzania and Benin revealed similar findings [33-36]. More recently, analysis of MAL-ED multi-country pooled data confirms the finding [37].

For peer review only - http://bmjopen.bmj.com/site/about/guidelines.xhtml

Our findings suggest that children living in crowded households (determined by number of people per room) were more likely to be stunted. This finding goes in line with a study done in Brazil which reported household people per room (OR 2.40; 95% CI: 1.36–4.22) to be associated with a high prevalence of stunting [38]. Another study done in Mexico showed that children living in crowded households (family with > 4 members) had a higher odds of being stunted than children living in families with 4 members or less [39]. Having a higher number of family members might have implications on household food insecurity or unavailability of complementary foods. The relationship between people per room at household level and prevalence of stunting demands further exploration keeping household food insecurity status as a covariate.

In longitudinal analysis, having separate room for kitchen was found to be negatively associated with stunting. Traditional cooking stoves are inefficient at combusting solid fuel and produces large volumes of indoor smoke [40]. Thus using traditional cooking materials inside the living room acts as a potent source of indoor air pollution by producing life threatening air pollutants [41]. This might have some biologically plausible relation with our finding. Multiple studies have reported air pollution to be associated with low birth weight [40 42]. However no such report was found about stunting and the variable showed near zero contribution in decomposition analysis.

Negative association between female gender and stunting needs some attention. A meta-analysis of 16 demographic and health surveys done in Sub-Saharan Africa reported boys to be more stunted than girls [43]. Similar results were found from East African herders where female biased parenting investments were reported to be the cause behind this [44]. But there is a paucity of data from South-Asian region that would echo our finding.

This study has limitations to report. Serum micronutrient data was collected at 7, 15 and 24 month. We had to carryforward 7 months data up to 14 month, 15 months data up to 23 months. 24 hour dietary

For peer review only - http://bmjopen.bmj.com/site/about/guidelines.xhtml

recall data collection started at 9 month. So to ensure the temporal direction of relation between outcome and explanatory variable we had to exclude the anthropometry data collected before 9th month.

CONCLUSION

The overall goal of the study was to measure the extent of selective determinants on stunting. We were focused to generate concrete evidence and to help the policy makers direct their programs on specific areas of life cycle to prevent stunting. The relation of maternal height and birth weight with childhood stunting affirms the intergenerational role and importance of proper adolescent nutrition. Programs should be directed for adolescent girls and women of reproductive age and adequate healthcare and nutrition must be provided. The goal of such community-based sustainable programs would be to improve the stature of adolescent girls as well as to improve the overall nutritional status of mothers during pregnancy that would have an impact on birth weight.

Acknowledgement: The authors would like to thank all the participants and their parents for sharing their time and providing consent, information and biological samples necessary for the successful completion of the study.

Contributions: SD and TA conceived the study. MAA managed the data set and provided technical support. SD analyzed the data, developed the tables/graphs and wrote the initial draft of the manuscript. All authors critically reviewed the manuscript and gave intellectual inputs.

Funding: This paper is made possible by the generous support of the American people through the United States Agency for International Development (USAID) under cooperative agreement no. AID-388-A-17-00006. The views expressed herein are responsibility of the RDM Activity and do not necessarily reflect the views of the U.S. Government or USAID or icddr,b, the implementing agency. The MAL-ED

For peer review only - http://bmjopen.bmj.com/site/about/guidelines.xhtml

(Etiology, Risk Factors, and Interactions of Enteric Infections and Malnutrition and the Consequences for Child Health) birth cohort study was funded by University of Virginia (UVA) with support from MAL-ED Network Investigators in the Foundation of National Institute of Health (FNIH), Fogarty International Centre (FIC) with overall support from the Bill & Melinda Gates Foundation (BMGF). They also acknowledge the contribution of icddr,b's core donors including Government of the People’s Republic of Bangladesh, Global Affairs Canada (GAC), Canada; Swedish International Development Cooperation Agency (SIDA) and the Department for International Development, UKAid for their continuous support and commitment to the icddr,b's research efforts.

Competing interests: The authors do not have any competing interests to declare.

Patient consent: Informed written consent was obtained from the mother or primary caregiver.

Data sharing statement: The data sets generated and/or analyzed during the current study is not made publicly available. However, data inquires or further suggestions for analyses can be made to the corresponding author.

REFERENCES

1. Pradhan M, Sahn DE, Younger SD. Decomposing world health inequality. *Journal of health economics* 2003;22(2):271-93.
2. Black RE, Allen LH, Bhutta ZA, et al. Maternal and child undernutrition: global and regional exposures and health consequences. *The lancet* 2008;371(9608):243-60.
3. Victora CG, Adair L, Fall C, et al. Maternal and child undernutrition: consequences for adult health and human capital. *The lancet* 2008;371(9609):340-57.
4. Grantham-McGregor S, Cheung YB, Cueto S, et al. Developmental potential in the first 5 years for children in developing countries. *The lancet* 2007;369(9555):60-70.
5. Global nutrition report 2017: nourishing the SDGs.
6. Adekanmbi VT, Uthman OA, Mudasiru OM. Exploring variations in childhood stunting in Nigeria using league table, control chart and spatial analysis. *BMC Public Health* 2013;13(1):361.
7. NIPORT M. Bangladesh Demographic and Health Survey BDHS 2014: Key Indicators. *Dhaka, Bangladesh and Calverton, Maryland, USA: National Institute of Population Research and Training (NIPORT), Mitra and Associates, and ICF International* 2014
8. National Institute of Population Research and Training (NIPORT) ICfDDR, Bangladesh (icddr,b), MEASURE Evaluation. Bangladesh Urban Health Survey 2013 Final Report.

For peer review only

For peer review only - http://bmjopen.bmj.com/site/about/guidelines.xhtml

9. Waterlow J. Introduction. Causes and mechanisms of linear growth retardation (stunting). *Eur J Clin Nutr* 1994;48(Suppl 1):S1-S4.

10. Hoddinott J, Alderman H, Behrman JR, et al. The economic rationale for investing in stunting reduction. *Maternal & Child Nutrition* 2013;9(S2):69-82.

11. Bhutta ZA, Ahmed T, Black RE, et al. What works? Interventions for maternal and child undernutrition and survival. *The lancet* 2008;371(9610):417-40.

12. Horton S, Shekar M, McDonald C, et al. Scaling up nutrition: What will it cost?: World Bank Publications 2009.

13. Ahmed T, Mahfuz M, Islam MM, et al. The MAL-ED cohort study in Mirpur, Bangladesh. *Clinical Infectious Diseases* 2014;59(suppl_4):S280-S86.

14. Lohman TG, Roche AF, Martorell R. Anthropometric standardization reference manual: Human kinetics books Champaign 1988.

15. WHO M. Growth, Reference, Study, Group. *WHO Child Growth Standards: Length/height-for-age, weight-for-age, weight-for-length, weight-for-height and body mass index-for-age: Methods and development Geneva: World Health Organization* 2006

16. Caulfield LE, Bose A, Chandyo RK, et al. Infant feeding practices, dietary adequacy, and micronutrient status measures in the MAL-ED study. *Clinical Infectious Diseases* 2014;59(suppl_4):S248-S54.

17. Daelmans B, Dewey K, Arimond M. New and updated indicators for assessing infant and young child feeding. *Food and nutrition bulletin* 2009;30(2_suppl2):S256-S62.

18. Psaki SR, Seidman JC, Miller M, et al. Measuring socioeconomic status in multicountry studies: results from the eight-country MAL-ED study. *Population health metrics* 2014;12(1):8.

19. Organization WH. Iron deficiency anaemia: assessment, prevention and control: a guide for programme managers. 2001

20. Thurnham DI, McCabe LD, Haldar S, et al. Adjusting plasma ferritin concentrations to remove the effects of subclinical inflammation in the assessment of iron deficiency: a meta-analysis–. *The American journal of clinical nutrition* 2010;92(3):546-55.

21. UNICEF B. National Micronutrients Status Survey. 2013

22. Baqui AH, Black RE, Yunus M, et al. Methodological issues in diarrhoeal diseases epidemiology: definition of diarrhoeal episodes. *International journal of epidemiology* 1991;20(4):1057-63.

23. Morris SS, Cousens SN, Lanata CF, et al. Diarrhoea—defining the episode. *International Journal of Epidemiology* 1994;23(3):617-23.

24. Lanata CF, Rudan I, Boschi-Pinto C, et al. Methodological and quality issues in epidemiological studies of acute lower respiratory infections in children in developing countries. *International journal of epidemiology* 2004;33(6):1362-72.

25. Pio A. Standard case management of pneumonia in children in developing countries: the cornerstone of the acute respiratory infection programme. *Bulletin of the World Health Organization* 2003;81(4):298-300.

26. Richard SA, Barrett LJ, Guerrant RL, et al. Disease surveillance methods used in the 8-site MAL-ED cohort study. *Clinical Infectious Diseases* 2014;59(suppl_4):S220-S24.

27. Diggle P, Liang K, Zeger S. Analysis of Longitudinal Data. Clarendon, Press: Oxford, 1994.

28. Sinning M, Hahn M, Bauer TK. The Blinder-Oaxaca decomposition for nonlinear regression models. *The Stata Journal* 2008;8(4):480-92.

29. Carey VJ, Lumley T, Ripley B. gee: Generalized Estimation Equation solver, R package version 4.13-18, 2015.

30. Hlavac M. oaxaca: Blinder-Oaxaca Decomposition in R. 2014

31. Ízaltin E, Hill K, Subramanian S. Association of maternal stature with offspring mortality, underweight, and stunting in low-to middle-income countries. *Jama* 2010;303(15):1507-16.

For peer review only - http://bmjopen.bmj.com/site/about/guidelines.xhtml

32. Hart N. Famine, maternal nutrition and infant mortality: a re-examination of the Dutch hunger winter. *Population Studies* 1993;47(1):27-46.
33. Nkurunziza S, Meessen B, Korachais C. Determinants of stunting and severe stunting among Burundian children aged 6-23 months: evidence from a national cross-sectional household survey, 2014. *BMC pediatrics* 2017;17(1):176.
34. Tiwari R, Ausman LM, Agho KE. Determinants of stunting and severe stunting among under-fives: evidence from the 2011 Nepal Demographic and Health Survey. *BMC pediatrics* 2014;14(1):239.
35. Chirande L, Charwe D, Mbwana H, et al. Determinants of stunting and severe stunting among under-fives in Tanzania: evidence from the 2010 cross-sectional household survey. *BMC pediatrics* 2015;15(1):165.
36. Padonou G, Le Port A, Cottrell G, et al. Factors associated with growth patterns from birth to 18 months in a Beninese cohort of children. *Acta tropica* 2014;135:1-9.
37. Investigators M-EN. Childhood stunting in relation to the pre-and postnatal environment during the first 2 years of life: The MAL-ED longitudinal birth cohort study. *PLoS medicine* 2017;14(10):e1002408.
38. Assis AMdO, Prado MdS, Barreto ML, et al. Childhood stunting in Northeast Brazil: the role of Schistosoma mansoni infection and inadequate dietary intake. *European Journal of Clinical Nutrition* 2004;58(7):1022.
39. Reyes H, Pérez-Cuevas R, Sandoval A, et al. The family as a determinant of stunting in children living in conditions of extreme poverty: a case-control study. *BMC Public Health* 2004;4(1):57.
40. Haider MR, Rahman MM, Islam F, et al. Association of low birthweight and indoor air pollution: Biomass fuel use in Bangladesh. *Journal of Health and Pollution* 2016;6(11):18-25.
41. Oliveira BFAd, Ignotti E, Hacon SS. A systematic review of the physical and chemical characteristics of pollutants from biomass burning and combustion of fossil fuels and health effects in Brazil. *Cadernos de saude publica* 2011;27(9):1678-98.
42. Chen L, Yang W, Jennison BL, et al. Air pollution and birth weight in northern Nevada, 1991-1999. *Inhalation toxicology* 2002;14(2):141-57.
43. Wamani H, Åstrøm AN, Peterson S, et al. Boys are more stunted than girls in sub-Saharan Africa: a meta-analysis of 16 demographic and health surveys. *BMC pediatrics* 2007;7(1):17.
44. Cronk L. Low Socioeconomic Status and Female-Biased Parental Investment: The Mukogodo Example. *American Anthropologist* 1989;91(2):414-29.

TABLES

Table 1: Maternal-, child-, household-level characteristics and morbidity status of the cohort

n=211 (at 24 month)	All (n=211)	Stunted (n=101)	Not stunted (n=110)
		n (%)	
Maternal-, child- and household-level characteristics			

For peer review only

For peer review only - http://bmjopen.bmj.com/site/about/guidelines.xhtml

Sex	Male	108 (51.2)	57 (56.4)	51 (46.4)
Exclusive breast feeding (WHO definition)	Yes	23 (10.9)	12 (11.9)	11 (10.0)
Household head's sex	Male	195 (92.4)	95 (94.1)	100 (90.9)
Mother's age	<18y	3 (1.4)	0 (0.0)	3 (2.7)
	18-30y	178 (84.4)	84 (83.2)	94 (85.5)
	>30y	30 (14.2)	17 (16.8)	13 (11.8)
Mother's age at first pregnancy	<18y	84 (39.8)	34 (33.7)	50 (45.5)
Maternal education	No education	41 (19.4)	22 (21.8)	19 (17.3)
	<5y	93 (44.1)	43 (42.6)	50 (45.5)
	>5y	77 (36.5)	36 (35.6)	41 (37.3)
Separate room for a kitchen	Yes	17 (8.1)	4 (4.0)	13 (11.8)

For peer review only - http://bmjopen.bmj.com/site/about/guidelines.xhtml

Monthly family income	<100 USD	94 (44.5)	52 (51.5)	42 (38.2)
Households have chicken or ducks	Yes	13 (6.2)	5 (5.0)	8 (7.3)
Treat water to make it safe	Yes	135 (64.0)	56 (55.4)	78 (70.9)
			Mean (SD)	
WAMI score*		0.53 (0.13)	0.50 (0.13)	0.55 (0.13)
People per room		3.57 (1.18)	3.83 (1.32)	3.33 (0.98)
Birth weight (kg)		2.82 (0.42)	2.70(0.39)	2.92(0.42)
Maternal height (cm)		149.03 (5.05)	147.34 (4.85)	150.54 (4.76)
	Morbidity episodes/child-year			
Diarrhea		3.63	4.03	3.43
ALRI		0.47	0.54	0.46
Fever		8.06	8.78	7.87
Cough		9.69	9.64	9.83

* The WAMI score (ranging from 0 to 1) is a measure of household socioeconomic status including access to improved Water, sanitation and hygiene (WASH), Assets, Maternal education, and Income.

Table 2: Determinants of stunting among children: results of Generalized Estimating Equation modeling

	Unadjusted odds (95% CI)	p-value	Adjusted odds (95% CI)	p-value
**Energy intake***	**1.001 (1.001 -1.001)**	**0.01**	**1.001 (1.001-1.002)**	**<0.00**
Ebf days	1.002 (1.001 - 1.003)	<0.00	1.002 (1.002-1.002)	0.81

For peer review only - http://bmjopen.bmj.com/site/about/guidelines.xhtml

Optimal IYCF	0.74 (0.85 - 0.64)	<0.00	0.89 (1.09-0.72)	0.25
Cough episodes	0.90 (0.98 - 0.82)	<0.00	0.97 (1.10-0.86)	0.67
Anemia	1.45 (1.69 - 1.26)	<0.00	1.09 (1.32-0.90)	0.40
**Gender (Female)**	**0.70 (0.80 - 0.61)**	**<0.00**	**0.47 (0.58-0.38)**	**<0.00**
**Maternal height***	**0.89 (0.91 - 0.88)**	**<0.00**	**0.87 (0.89-0.86)**	**<0.00**
**Separate room for a kitchen**	**0.37 (0.50 - 0.28)**	**<0.00**	**0.21 (0.38-0.12)**	**<0.00**
WAMI	0.06 (0.11 - 0.04)	<0.00	0.39 (1.03-0.15)	0.06
**People per room***	**1.34 (1.42 - 1.26)**	**<0.00**	**1.48 (1.62-1.35)**	**<0.00**
**Birth weight***	**0.18 (0.22 - 0.15)**	**<0.00**	**0.11 (0.15-0.09)**	**<0.00**
Serum Zinc	0.94 (0.98 - 0.91)	<0.00	0.99 (1.04-0.95)	0.79

*per one unit change

Table 3: Relative contribution of the selected variables on mean length-for-age Z score difference between stunted and non-stunted children at 24 month

	Endowment			Coefficient			Interaction		
	Coef.	95% CI		Coef.	95% CI		Coef.	95% CI	
		Upper	Lower		Upper	Lower		Upper	Lower
Overall	0.26	0.41	0.10	1.34	1.50	1.19	-0.12	0.03	-0.26

For peer review only - http://bmjopen.bmj.com/site/about/guidelines.xhtml

Energy intake	0.00	0.03	-0.03	-0.12	0.22	-0.46	0.00	0.03	-0.03
Gender	0.01	0.02	-0.01	0.02	0.11	-0.07	0.00	0.01	-0.02
Maternal height	0.10	0.18	0.02	-3.04	0.35	-6.44	-0.07	0.02	-0.15
Separate kitchen	0.00	0.03	-0.03	0.01	0.03	-0.02	0.02	0.06	-0.03
People per room	0.06	0.12	0.00	0.31	0.92	-0.30	-0.04	0.04	-0.12
Birth weight	0.08	0.17	-0.01	-0.21	0.88	-1.30	-0.02	0.07	-0.11

Figure legends

Figure 1

Title: Patterns of macronutrient intake from 9 to 24 months

Legends: Mean macronutrient intake per day, Months, Animal protein, Carbohydrate, Fat, Total protein, Stunted, Not stunted

Figure 2

Title: Prevalence of stunting among children who did not take Minimum Acceptable Diet (MAD) and those who took it

Legends: Months, Did not take MAD, Took MAD

Supplementary figure 1

Title: Serum zinc, ferritin and hemoglobin levels of stunted and non-stunted children at 7, 15, and 24 months

Legends: Zinc, Ferritin, Hemoglobin, Stunted, No, Yes

Supplementary figure 2

Title: Relative contribution of the selected variables on mean Length for Age Z score difference between stunted and non-stunted children at 24 months

For peer review only

For peer review only - http://bmjopen.bmj.com/site/about/guidelines.xhtml

Legends: Endowments, Coefficients, Interaction, Energy intake, Gender, Maternal height, Separate kitchen, Crowding, Birth weight

For peer review only

For peer review only - http://bmjopen.bmj.com/site/about/guidelines.xhtml

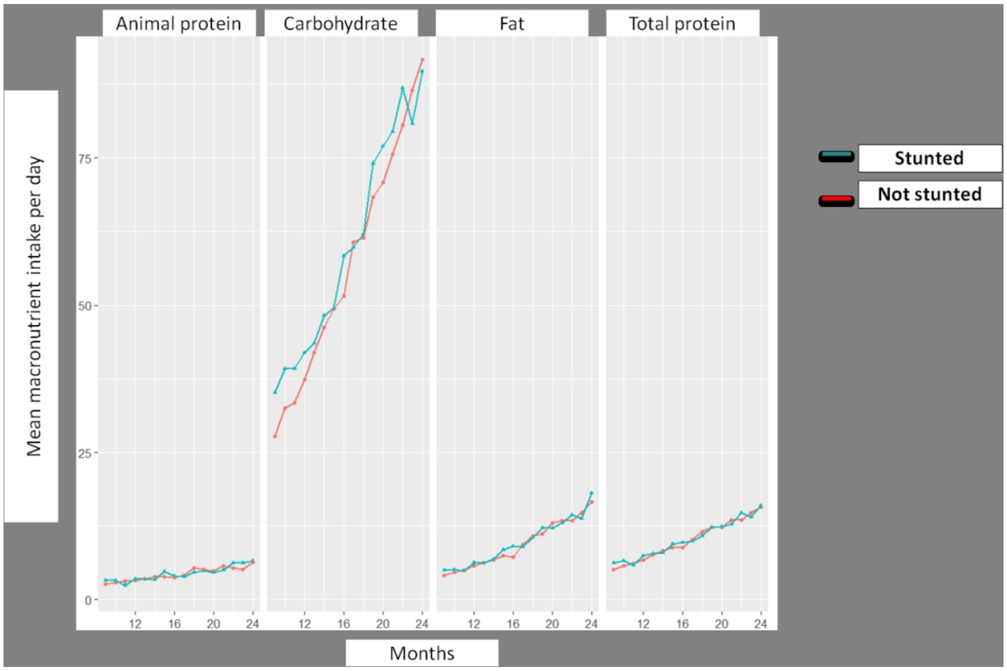

Caption: Patterns of macronutrient intake from 9 to 24 months
Legends: Mean macronutrient intake per day, Months, Animal protein, Carbohydrate, Fat, Total protein, Stunted, Not stunted

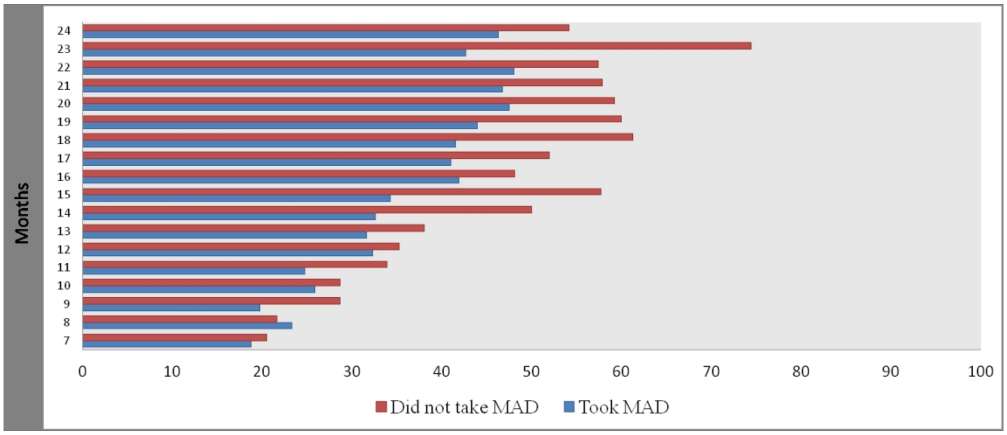

Caption: Prevalence of stunting among children who did not take Minimum Acceptable Diet (MAD) and those who took it
Legends: Months, Did not take MAD, Took MAD

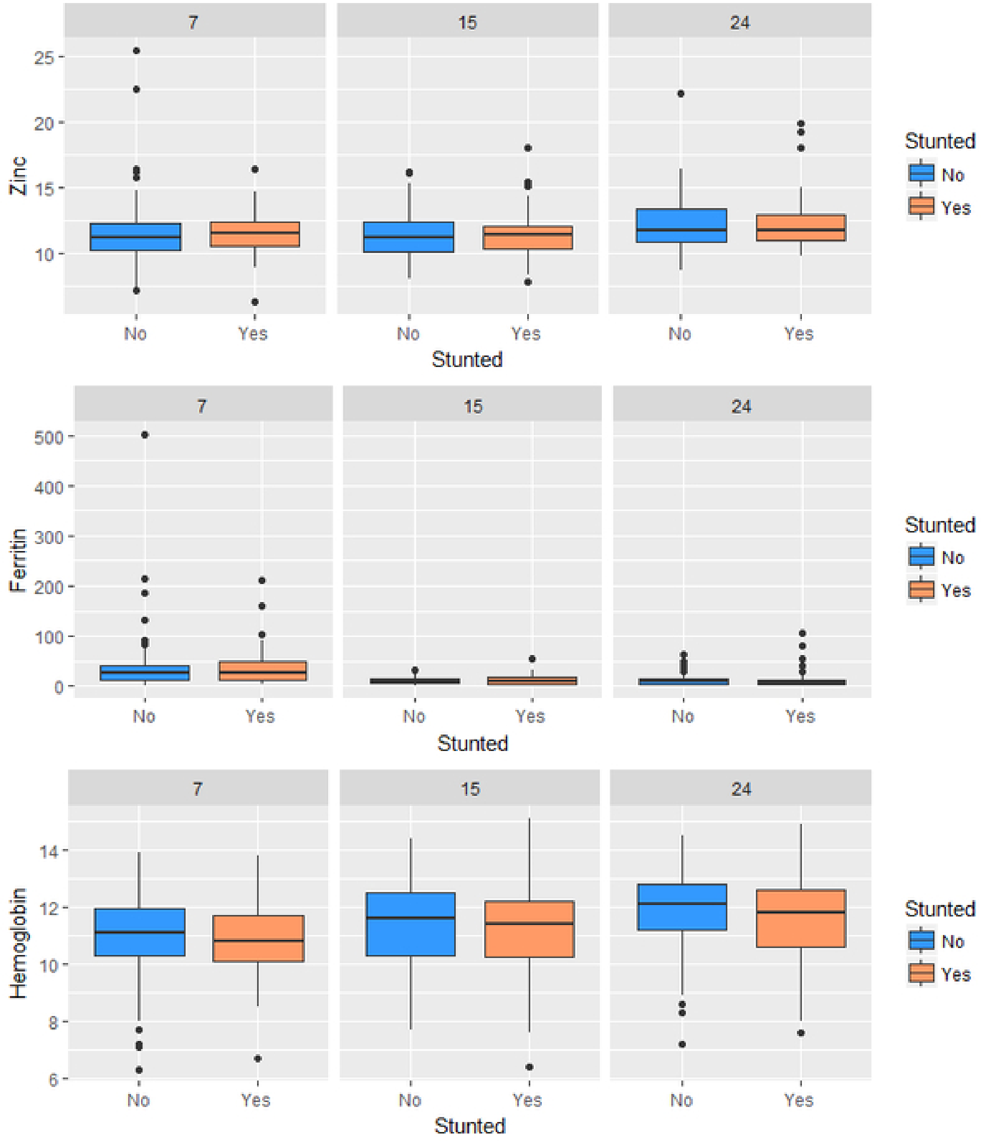

Caption: Serum zinc, ferritin and hemoglobin levels of stunted and non-stunted children at 7, 15, and 24 months
Legends: Zinc, Ferritin, Hemoglobin, Stunted, No, Yes

51x58mm (300 x 300 DPI)

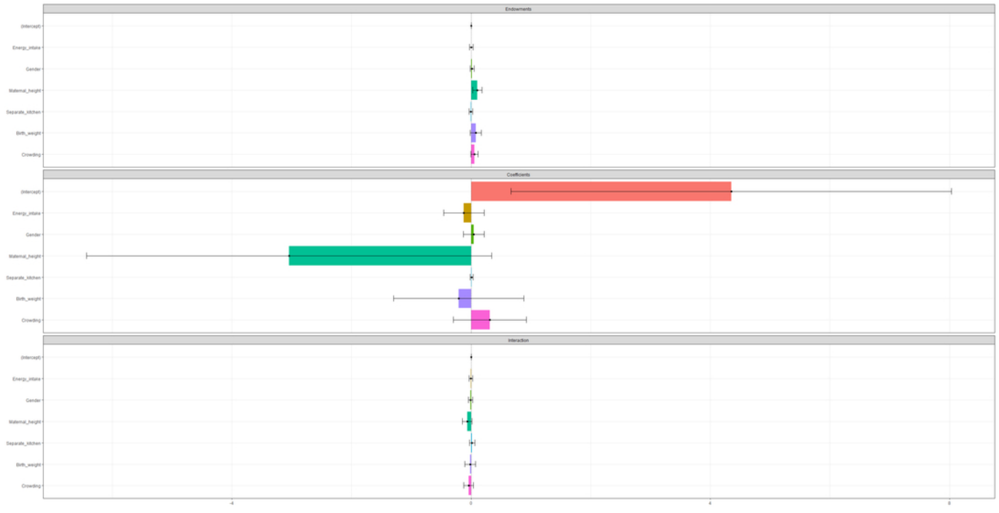

Caption: Relative contribution of the selected variables on mean Length for Age Z score difference between stunted and non-stunted children at 24 months
Legends: Endowments, Coefficients, Interaction, Energy intake, Gender, Maternal height, Separate kitchen, Crowding, Birth weight

92x48mm (300 x 300 DPI)

STROBE (Strengthening The Reporting of OBservational Studies in Epidemiology) Checklist

A checklist of items that should be included in reports of observational studies. You must report the page number in your manuscript where you consider each of the items listed in this checklist. If you have not included this information, either revise your manuscript accordingly before submitting or note N/A.

Note: An Explanation and Elaboration article discusses each checklist item and gives methodological background and published examples of transparent reporting. The STROBE checklist is best used in conjunction with this article (freely available on the Web sites of PLoS Medicine at http://www.plosmedicine.org/, Annals of Internal Medicine at http://www.annals.org/, and Epidemiology at http://www.epidem.com/). Information on the STROBE Initiative is available at www.strobe-statement.org.

Section and Item	Item No.	Recommendation	Reported on Page No.
**Title and Abstract**	1	(*a*) Indicate the study's design with a commonly used term in the title or the abstract	1,2
		(*b*) Provide in the abstract an informative and balanced summary of what was done and what was found	1,2
**Introduction**			
Background/Rationale	2	Explain the scientific background and rationale for the investigation being reported	3-4
Objectives	3	State specific objectives, including any prespecified hypotheses	4
**Methods**			
Study Design	4	Present key elements of study design early in the paper	4
Setting	5	Describe the setting, locations, and relevant dates, including periods of recruitment, exposure, follow-up, and data collection	4
Participants	6	(*a*) *Cohort study*—Give the eligibility criteria, and the sources and methods of selection of participants. Describe methods of follow-up	4
		Case-control study—Give the eligibility criteria, and the sources and methods of case ascertainment and control selection. Give the rationale for the choice of cases and controls	n/a
		Cross-sectional study—Give the eligibility criteria, and the sources and methods of selection of participants	n/a
		(*b*) *Cohort study*—For matched studies, give matching criteria and number of exposed and unexposed	n/a
		Case-control study—For matched studies, give matching criteria and the number of controls per case	n/a
Variables	7	Clearly define all outcomes, exposures, predictors, potential confounders, and effect modifiers. Give diagnostic criteria, if applicable	5-6

For peer review only

Section and Item	Item No.	Recommendation	Reported on Page No.
Data Sources/ Measurement	8*	For each variable of interest, give sources of data and details of methods of assessment (measurement). Describe comparability of assessment methods if there is more than one group	5-6
Bias	9	Describe any efforts to address potential sources of bias	7
Study Size	10	Explain how the study size was arrived at	4
Quantitative Variables	11	Explain how quantitative variables were handled in the analyses. If applicable, describe which groupings were chosen and why	7
Statistical Methods	12	(*a*) Describe all statistical methods, including those used to control for confounding	7
		(*b*) Describe any methods used to examine subgroups and interactions	n/a
		(*c*) Explain how missing data were addressed	7
		(*d*) *Cohort study*—If applicable, explain how loss to follow-up was addressed	7
		Case-control study—If applicable, explain how matching of cases and controls was addressed	n/a
		Cross-sectional study—If applicable, describe analytical methods taking account of sampling strategy	n/a
		(*e*) Describe any sensitivity analyses	n/a
**Results**			
Participants	13*	(a) Report numbers of individuals at each stage of study—eg numbers potentially eligible, examined for eligibility, confirmed eligible, included in the study, completing follow-up, and analysed	7
		(b) Give reasons for non-participation at each stage	n/a
		(c) Consider use of a flow diagram	n/a
Descriptive Data	14*	(a) Give characteristics of study participants (eg demographic, clinical, social) and information on exposures and potential confounders	7,8
		(b) Indicate number of participants with missing data for each variable of interest	n/a
		(c) *Cohort study*—Summarise follow-up time (eg, average and total amount)	4
Outcome Data	15*	*Cohort study*—Report numbers of outcome events or summary measures over time	7
		Case-control study—Report numbers in each exposure category, or summary measures of exposure	
		Cross-sectional study—Report numbers of outcome events or summary measures	

For peer review only

Section and Item	Item No.	Recommendation	Reported on Page No.
Main Results	16	(*a*) Give unadjusted estimates and, if applicable, confounder-adjusted estimates and their precision (eg, 95% confidence interval). Make clear which confounders were adjusted for and why they were included	9,17
		(*b*) Report category boundaries when continuous variables were categorized	n/a
		(*c*) If relevant, consider translating estimates of relative risk into absolute risk for a meaningful time period	n/a
Other Analyses	17	Report other analyses done—eg analyses of subgroups and interactions, and sensitivity analyses	n/a
**Discussion**			
Key Results	18	Summarise key results with reference to study objectives	10,11
Limitations	19	Discuss limitations of the study, taking into account sources of potential bias or imprecision. Discuss both direction and magnitude of any potential bias	11
Interpretation	20	Give a cautious overall interpretation of results considering objectives, limitations, multiplicity of analyses, results from similar studies, and other relevant evidence	10,11
Generalisability	21	Discuss the generalisability (external validity) of the study results	10,11
**Other Information**			
Funding	22	Give the source of funding and the role of the funders for the present study and, if applicable, for the original study on which the present article is based	12

*Give information separately for cases and controls in case-control studies and, if applicable, for exposed and unexposed groups in cohort and cross-sectional studies.

Once you have completed this checklist, please save a copy and upload it as part of your submission. DO NOT include this checklist as part of the main manuscript document. It must be uploaded as a separate file.

For peer review only

BMJ Open

Relative contributions of the correlates of stunting in explaining the mean length-for-age z-score difference between 24-month-old stunted and non-stunted children living in a slum of Dhaka, Bangladesh: Results from a decomposition analysis

Journal:	*BMJ Open*
Manuscript ID	bmjopen-2018-025439.R1
Article Type:	Research
Date Submitted by the Author:	18-Feb-2019

Complete List of Authors:	Das, Subhasish; International Centre for Diarrhoeal Disease Research, Bangladesh (icddr,b), Nutrition and Clinical Services Division; Employer<br>Alam, Md Ashraful; International Centre for Diarrhoeal Disease Research Bangladesh, Nutrition and Clinical Services Division<br>Mahfuz, Mustafa; International Centre for Diarrhoeal Disease Research, Nutrition and Clinical Services Division<br>Arifeen, Shams E.; ICDDR B, Maternal and Child Health Division (MCHD)<br>Ahmed, Tahmeed; International Centre for Diarrhoeal Disease Research, Bangladesh (icddr,b), Nutrition and Clinical Services Division (NCSD)

**Primary Subject Heading**:	Public health
Secondary Subject Heading:	Epidemiology, Global health, Nutrition and metabolism, Public health
Keywords:	Relative contribution, Decomposition analysis, Maternal height, Stunting, Length-for-age z-score

SCHOLARONE™ Manuscripts

TITLE: Relative contributions of the correlates of stunting in explaining the mean length-for-age z-score difference between 24-month-old stunted and non-stunted children living in a slum of Dhaka, Bangladesh: Results from a decomposition analysis

Corresponding author:

Subhasish Das, Nutrition and Clinical Services Division, International Centre for Diarrhoeal Disease Research Bangladesh (icddr,b), Dhaka 1212, Bangladesh. Email: subhasish.das@icddrb.org. Telephone number: +8801617099766

Co-authors:

Md Ashraful Alam, Nutrition and Clinical Services Division, icddr,b, Dhaka, Bangladesh

Mustafa Mahfuz, Nutrition and Clinical Services Division, icddr,b, Dhaka, Bangladesh

Shams El Arifeen, Maternal and Child Health Division, icddr,b, Dhaka, Bangladesh

Tahmeed Ahmed, Nutrition and Clinical Services Division, icddr,b, Dhaka, Bangladesh

Authors' email addresses: subhasish.das@icddrb.org, mashraful@icddrb.org, mustafa@icdddrb.org, shams@icddrb.org, tahmeed@icddrb.org

Body text word count: 3471

Keywords: Stunting; Length-for-age z-score; Relative contribution; Decomposition analysis; Maternal height.

ABSTRACT

For peer review only - http://bmjopen.bmj.com/site/about/guidelines.xhtml

Objective: Using Mal-ED Bangladesh birth cohort data we sought to measure the relative contributions of the most predictive correlates of stunting to mean length-for-age z (LAZ) score difference between stunted and non-stunted children at 24 months of age.

Setting: Dhaka, Bangladesh

Participants: 211 slum dwelling children enrolled within 17 days of their birth.

Variables and method: The explanatory variables were identified from following groups: maternal characteristics, birth characteristics, macronutrient intake, socio-economic status, morbidity and serum micronutrient level. At step 1, predictive correlates of stunting were identified longitudinally (from 9 to 24 months of age) using generalized estimating equations (GEE) model. Then, the relative contributions of the most predictive correlates of stunting to mean LAZ score difference between stunted and non-stunted children at 24 months of age was measured using Blinder-Oaxaca decomposition analysis.

Results: The GEE multivariable model identified maternal height, birth weight, people per room, gender, having separate room for kitchen and energy intake as the most predictive correlates of stunting. At 24 months, mean LAZ score difference between stunted and non-stunted children was 1.48. The variable by variable decomposition of the LAZ gap identified maternal height (coefficient: -3.04 ; 95% CI: 0.35, -6.44), birth weight (coefficient: -0.21; 95% CI: 0.88, -1.30), people per room (coefficient: 0.31; 95% CI: 0.92, -0.30) and energy intake (coefficient: -0.12; 95% CI: 0.22, -0.46) as the top most factors responsible for the mean LAZ score difference between stunted and non-stunted children at 24 months of age.

Conclusions: The relative contributions of maternal height and birth weight to LAZ gap signifies that improvement in nutritional status of a women during her adolescence and pregnancy would have an impact on birth weight of her offspring, and ultimately, on linear growth of the child.

For peer review only

For peer review only - http://bmjopen.bmj.com/site/about/guidelines.xhtml

Strengths and limitations of this study

- This study used the threefold Blinder-Oaxaca decomposition analysis to decompose the relative contributions of most predictive correlates of stunting to mean LAZ score difference of stunted and non-stunted children into three components: endowments, coefficients, and the interaction of the two.
- The explanatory variables used for the decomposition analysis were identified after analyzing the longitudinal birth cohort data collected from the same children.
- At 24 months, 20% of the enrolled children were lost to follow-up which could lead to possible bias.
- The findings of this study might not be applicable to the non-slum setting, as this study was conducted in a slum area of Dhaka, Bangladesh.

INTRODUCTION

Stunting (length-for-age more than two standard deviations below the WHO Child Growth Standards median) is an indicator of chronic undernutrition and is one of the best measures of child health inequality.[1] It is one of the most prevalent forms of malnutrition which is known to be associated with higher mortality and cognitive impairment of affected children.[1-4] Worldwide, 150.8 million under 5 children are stunted and in Asia, the prevalence is 23.2%.[5] Variation in rates of stunting exists not only across continents but also within local geographies.[6] The prevalence of stunting in Bangladeshi slum areas is much higher than the national level average (50 vs. 36 %, respectively).[7 8]

Children from lowest wealth quintiles who have a normal anthropometric status at birth may experience an intense period of growth retardation.[9] During the subsequent life-cycle stages this growth

For peer review only - http://bmjopen.bmj.com/site/about/guidelines.xhtml

retardation continues to exert negative effects such as socio-emotional impairment in pre-school age, poor school attainment in early childhood, and higher risk of chronic disease in adulthood.[10] WHO conceptual framework on childhood stunting reported maternal factors, home environment, inadequate complementary feeding, breast feeding and infection as the most proximal factors responsible for stunting.[11] But, relative contributions of these correlates to length-for-age z (LAZ) score difference between stunted and non-stunted children were not measured. In high burden countries the benefit-cost ratio of investments to reduce stunting ranges from 3.8 to 47.9, whereas the ratio is 18.4 in Bangladesh.[12] To that end, since Bangladesh is a resource constrained country, identifying the top contributors of stunting would help the policy-makers to direct concerted stunting prevention programs in a cost effective way. Keeping the context in mind, we identified the correlates of stunting among 9-24 months old children using the longitudinal data emanated from a birth cohort study. Then, we measured the relative contributions of the most predictive correlates of stunting in explaining the mean LAZ score difference between stunted and non-stunted children at 24 months of age.

METHODS

Study participants

Data used for this analysis was collected from the MAL-ED (Etiology, Risk Factors, and Interactions of Enteric Infections and Malnutrition and the Consequences for Child Health) Bangladesh site. The MAL-ED birth cohort study was conducted at 8 sites in 3 continents. In Bangladesh the MAL-ED study site was Bauniabadh slum, Mirpur, Dhaka which was described elsewhere.[13] Following a well-defined protocol, field workers visited households and enrolled 265 healthy newborn children within 17 days of their birth between February 2010 and February 2012. Participants were followed till 24 months of age. At 24 months, 211 participants remained which is 80% of the total recruitment. The study was approved by the Institutional Review Board of icddr,b (protocol no. PR-2008-020).

For peer review only - http://bmjopen.bmj.com/site/about/guidelines.xhtml

Data collection

Dependent variable

Following standard anthropometric methodology trained field workers measured the recumbent length of the children to the nearest 1 mm using Seca infantometer (model no.417, Hamburg, Germany) every month until 24 months of age.[14] The LAZ score of each child was determined using the WHO 2006 child growth standards.[15] A child with LAZ < –2 is classified as stunted.[15]

Independent variables

The explanatory variables used for the analysis were drawn from the following groups: birth characteristics, maternal characteristics, nutrient intake, socio-economic status, morbidity and serum micronutrient. Data on birth characteristics (enrollment weight and length) and maternal height were collected within 72 hours of recruitment. Children, wearing minimum cloths, were weighed using a digital scale with 10 g precision (Seca, model no.345, Hamburg, Germany). The enrollment weight was used as the surrogate measure for birth weight. Standard wooden height measuring boards were used for measuring maternal height. For assessing the feeding habit and energy intake of the children, 24-hour food frequency data was collected monthly from 9th month onwards using a 24-hour multiple-pass dietary recall approach.[16] The 24-hour dietary recall interviews were conducted on non-consecutive days and out of every four recalls, one interview was conducted on a weekend. A locally adapted food composition table was used to convert the dietary intake data to energy.[17] Minimum acceptable diet (MAD)- a core indicator of infant and young child feeding practice was used to measure the appropriateness of the complementary feeding practice of the children.[18] MAD meets standards for both minimum dietary diversity and minimum meal frequency. Socio-economic data was collected at 6, 12, 18 and 24 months. WAMI index (ranging from 0 to 1) -a socio-economic status index which includes

For peer review only - http://bmjopen.bmj.com/site/about/guidelines.xhtml

access to improved water and sanitation, eight selected assets, maternal education, and household income was used as a representative of socio-economic status of the households.[19] To measure the household level crowding a continuous variable named "people per room" was generated by dividing the total number of people usually slept in the household by the number of rooms of that household used for sleeping. Blood samples for measuring serum zinc, ferritin and hemoglobin status were collected at 7, 15 and 24 months of age of a child. For hemoglobin, capillary blood was collected and measured with the Hemocue device (Hb 201, Ängelholm, Sweden). Plasma zinc and ferritin levels were measured using atomic absorption spectometry (AAS) and chemiluminescence immunoassay (CLIA), respectively. Anemia is defined as a hemoglobin concentration <110 g/L.[20] Plasma ferritin and zinc concentration values were adjusted for the effect of subclinical inflammation using both C-reactive protein (CRP) and α-1-acid glycoprotein (AGP).[21]

Trained field workers visited the participant's household twice a week and used a surveillance assessment form (SAF) to document the breast feeding and illness status of the child. Household visits were generally made on every 3–4 days. During every such visits, field workers asked the caregiver whether the children experienced any of the symptoms listed in SAF on each day since the last visit.[22] To document the breastfeeding status, the staff asked the mother about the liquids the child consumed during the past 24 hours. If the response followed the WHO definition of exclusive breast feeding (no other food or drink, not even water, except breast milk (including milk expressed, ORS, drops and vitamins, minerals and medicines syrups)), then the child was considered as exclusively breast fed. Diarrhea is defined as having ≥3 loose stools in a 24-hour period or at least 1 loose stool with blood reported by the mother. [23] A diarrheal episode is defined as being separated from another episode by at least 2 or more diarrhea-free days.[23-25] Acute lower respiratory infection (ALRI) is identified based on the presence of cough and/or shortness of breath plus high respiratory rate.[25] The average of two respiratory rates was used, and was considered high based on the age of the child (<2 months of age:

For peer review only - http://bmjopen.bmj.com/site/about/guidelines.xhtml

129 ≥60 breaths/minute; 2 to <12 months of age: ≥50 breaths/minute; ≥12 months of age: ≥40 breaths/minute).[26] ALRI episodes are separated by at least 15 ALRI-free days. [25] Fever is defined as an axillary temperature >37.5°C. [22]

132 **Statistics**

133 We reported the maternal-, child-, and household-level characteristics of the study participants using mean and standard deviation for continuous variables and frequency and percentage for categorical variables. Student's t-test, Chi-square test or the Fisher's exact test was used to compare the above mentioned characteristics of the stunted children to their non-stunted counterparts at 24 months of age.

138 At stage 1, we analyzed the longitudinal birth cohort data to identify the most predictive correlates of stunting. Serum micronutrient data was collected at 7, 15 and 24 months of age of the children. We carried forward 7 months data up to 14 months and 15 months data up to 23 months. Dietary intake data collection started at 9 months of age. Hence, to ensure the availability of maximum variables at every time point and temporality of the relation between outcome and explanatory variables, the longitudinal data analysis was done using the data collected from 9 to 24 months of age. Instead of exclusive breast feeding (ebf) status (yes vs. no), exclusive breast feeding days (ebf days) was used as it counts the specific number of days. The association between each explanatory variable and the binary outcome variable (stunting: no vs. yes) was examined longitudinally using population specific generalized estimation equations (GEE).[27] The generalized estimating equations (GEE) method is an extension of the quasi-likelihood approach that permits specification of a working correlation matrix for the within-subject correlation of repeated responses collected from the same participants over time. As a result, more efficient and unbiased regression parameters are produced. Initially, bivariate analyses were performed to identify the unadjusted effect of each explanatory variable on the outcome variable.

For peer review only - http://bmjopen.bmj.com/site/about/guidelines.xhtml

The explanatory variables showing statistically significant association (p value <0.05) with the outcome variable in the bivariate analyses were selected for multivariable modeling. The variance inflation factor (VIF) was calculated to detect multicollinearity and variables producing a VIF value > 5 were excluded from the analysis. Quasi-likelihood information criterion (QIC) was used to select the correct covariance structure. The multivariable model with unstructured covariance matrix produced the smallest QIC value. Hence, we report the results of the multivariable model that was constructed using unstructured covariance matrix with robust variance estimates. We determined the strength of association by estimating the odds ratios and their 95% confidence intervals. Statistical significance was determined at a probability value less than 0.05 and the variables showing statistically significant association with stunting in the multivariable GEE model were selected for the decomposition analysis.

At stage 2, threefold Blinder-Oaxaca decomposition analysis was done to measure the relative contribution of the identified variables to mean length-for-age z-score difference between stunted and non-stunted children at 24 months of age. The threefold decomposition analysis decomposes the gap or difference of the group-specific means into three components- a gap due to differences in the level or magnitude of determinants or endowments, a gap due to the differences in the effects of the determinants or coefficients, and a gap due to the interactions between endowments and coefficients.[28] During decomposition analysis bootstrapped standard errors were calculated based on 1,000 replicates. R version 3.4.2 was used for data analysis and GEE and Oaxaca packages were employed.[29 30]

Patient and Public Involvement (PPI) statement

No patient or public were involved with the development of research question, designing the study, recruitment of participants, interpretation of the results, and will be involved during disseminating the findings of the paper.

For peer review only - http://bmjopen.bmj.com/site/about/guidelines.xhtml

RESULTS

At month 1, the cohort exhibited a 19% prevalence of stunting. The prevalence showed a similar trend till month 6 (18.4 %). After that, this trend went up to 33.6%, 46.8% and 47.9% at 12, 18 and 24th months, respectively.

The socio-demographic characteristics of the participating households are described in table 1. Till 24 months, the study was able to follow a cohort of 211 children. At 24 months, the overall male to female ratio was 1.04, which was 1.3 in the stunted cohort. Only 10.9% of children were exclusively breastfed, the prevalence of which did not change over the stunting status. Most of the households were headed by a male family member. One-fifth of the mothers were illiterate and nearly half of them had an education of less than 5 years. Nearly half of the households had a monthly income of less than 100 USD, the percentage of which was higher in the stunted group than the non-stunted group. Mean birth weight of the stunted children was lower than their non-stunted counterparts. The mothers of the stunted children also had a lower height than those of the non-stunted children.

Morbidity data displayed in table 1 show that per year stunted children suffered from more than 4 diarrheal episodes which was higher than the overall annual rate of 3.63 episodes. Similar trends were seen for ALRI, fever and cough episodes.

Figure 1 represents the pattern of macronutrient intake from 9 to 24 months. Over the period, the trend of nutrient intake did not differ much between the stunted and non-stunted groups. For both the groups, carbohydrate was the main source of nutrition.

Figure 2 presents the comparative picture of prevalence of stunting among the children with and without minimum acceptable diet (MAD). Children without minimally acceptable diets were more

For peer review only - http://bmjopen.bmj.com/site/about/guidelines.xhtml

stunted than children with minimally acceptable diets, and from 9 months onwards the difference in prevalence of stunting between these two groups increased with age.

Box plots reported in supplementary figure 1 show the comparative picture of serum zinc, ferritin and hemoglobin level of stunted and non-stunted children at 7, 15, and 24 months. Bivariate GEE modeling revealed that, over the months, serum zinc and hemoglobin levels had statistically significant (p value <0.05) association with stunting.

Table 2 presents the results of generalized estimating equation modeling. Thorough literature review was done to identify the variables of interest and bivariate GEE modeling was carried out longitudinally by taking into account all the variables, one at a time. However, table 2 shows only those variables found to have a statistically significant association (p value <0.05) with stunting. From nutrient intake group the amount of energy intake was selected because separate food groups (carbohydrate, total protein, animal protein and fat) showed a very high correlation with each other. The multivariable model showed significant (p value < 0.05) association of energy intake, gender, maternal height, having a separate room for kitchen, people per room and birth weight with stunting.

Table 3 and supplementary figure 2 illustrate the results of Blinder-Oaxaca decomposition analysis. At month 24, the mean LAZ score of non-stunted group was -1.33 and in the stunted group it was -2.81, yielding a LAZ gap of 1.48. The stunted-non stunted LAZ gap had subsequently been decomposed into three components- a gap due to differences in the level or magnitude of determinants (endowments), a gap due to the differences in the effects of the determinants (coefficients) and a gap due to the interactions between endowments and coefficients. The results of the threefold decomposition suggest that, of the 1.48 difference, approximately 0.26 (95% CI: 0.41, 0.10) was due to the group differences in endowments, 1.34 (95% CI: 1.50, 1.19) to differences in coefficients, and the remaining -0.12 (95% CI: 0.03, -0.26) was for the interaction of the two. The ‘endowment’ part of the result reflects the average

For peer review only

For peer review only - http://bmjopen.bmj.com/site/about/guidelines.xhtml

increase in stunted children's LAZ if they had the same level of characteristics as non-stunted children. This also indicates that the differences in the magnitude of explanatory variables account for about 17.6% of the LAZ gap among stunted and non-stunted children. The coefficient part shows that if the effect of the non-stunted children's characteristics was applied to the stunted children, the changes in the LAZ of the stunted cohort would be 1.34. The interaction component indicates that the simultaneous effect of endowment and coefficient was -0.12. The findings suggest that the gap in the means of LAZ is primarily due to the coefficient or effect of the determinants. The variable by variable decomposition of the LAZ gap identified maternal height (coefficient: -3.04 ; 95% CI: 0.35, -6.44), birth weight (coefficient: -0.21; 95% CI: 0.88, -1.30), people per room (coefficient: 0.31; 95% CI: 0.92, -0.30) and energy intake (coefficient: -0.12; 95% CI: 0.22, -0.46) as the top most factors responsible for the mean length-for-age z score difference between stunted and non-stunted at 24 months of age.

DISCUSSION

At month 24 the cohort exhibited 47.9% prevalence of stunting. The figure is similar to Bangladesh Urban Health Survey which reported a 50% prevalence of stunting in under 5 children living in slum areas of Dhaka, Bangladesh.[8]

Our analysis found maternal height to be inversely associated with stunting and exerting the highest relative contribution to LAZ score difference between stunted and non-stunted children at 24 months of age. Comparable findings were reported from studies conducted in similar setting. Results from 109 Demographic and Health Surveys conducted in 54 LMICs between 1991 and 2008 found maternal stature to be inversely associated with stunting during infancy and childhood.[31] It has also been published that a child, born to a shorter mother, had greater chances of being in a lower length-for-age category.[32] This could be explained by the biological plausibility that shorter mothers may have a poorer

For peer review only - http://bmjopen.bmj.com/site/about/guidelines.xhtml

health and nutritional status and failed to deliver adequate nutrition to the fetus during pregnancy.[33] This finding signifies the intergenerational effect of malnutrition.

The negative association between birth weight and stunting identifies it as another important correlate of stunting. Moreover, the relative contribution of birth weight on mean length-for-age z score difference between stunted and non-stunted children at their 24 months of age indicates that its contribution did not decline with age. After birth, the mean length-for-age z score experiences a sharp fall, the magnitude of which can be affected by the presence of the growth hindering factor - low birth weight.[34 35] A meta-analysis of 19 studies showed that under 5 children with low birth weight had 3-fold higher odds of becoming stunted.[36]

Our findings suggest that children living in crowded households were more likely to be stunted. It also contributed to the mean LAZ sore difference between stunted and non-stunted children. This finding corresponds with the elucidations from a study done in Brazil which reported household people per room (OR 2.40; 95% CI: 1.36–4.22) to be associated with a high prevalence of stunting.[37] Another study conducted in India showed that children living in crowded households (family with ≥3 persons per room) had a higher odds of being stunted than the children living in families with 3 members or less.[38] Having a higher number of family members may raise implications on household food insecurity or unavailability of complementary foods. Hence, the relationship warrants a further exploration keeping household food insecurity status as a covariate.

Though energy intake showed a marginal association with stunting in longitudinal data analysis, it did contribute to the LAZ gap at 24 months. This finding is supported by the multicountry analysis of MAL-ED birth cohort data which reported that lower percent of energy from dietary protein was strongly associated with a decline in length-for-age.[32] Another study done in Zambia revealed that the stunted infants and toddlers had a tendency to take lower energy compared to the non-stunted children.[39]

For peer review only - http://bmjopen.bmj.com/site/about/guidelines.xhtml

We found that female children had lesser chances of becoming stunted than their male counterparts. A meta-analysis of 16 demographic and health surveys done in sub-Saharan Africa reported boys to be more stunted than girls.[40] Similar results were found from East African herders where parental investments were biased for their female children.[41] On the contrary, studies also reported that in sub-Saharan areas daughters faced a gender biased dietary discrimination that was in favor of boys.[42] A cross-sectional analysis done in Bangladesh, Nepal and India documented that parental preference towards son might affect child undernutrition in this region. [43] However, there is a paucity of data that could elaborate this relation and the variable showed an insubstantial contribution in decomposition analysis.

In longitudinal analysis, having a separate room for kitchen was found to be negatively associated with stunting. Traditional cooking stoves inefficiently combust solid fuels and produce large volumes of indoor smoke.[44] Thus using traditional cooking materials inside the living room acts as a potent source of indoor air pollution by producing life threatening air pollutants.[45] This might have some biologically plausible relation with our finding. Multiple studies have reported air pollution to be associated with low birth weight.[44 46] However, no such report was found regarding stunting and the variable showed near zero contribution in decomposition analysis.

In addition to identifying the determinants of stunting, our study provided additional unique findings that the coefficients or effects of those determinants were primarily responsible for the stunted vs. non stunted LAZ gap at 24 months of age. That is the strength of this analysis. But, this study also has its own limitations to report. At month 24, 20% of the enrolled children were lost to follow-up which can lead to potential attrition bias. Moreover, the study was conducted in a slum area. Hence, the findings of this study may not be applicable to the non-slum setting.

CONCLUSION

For peer review only - http://bmjopen.bmj.com/site/about/guidelines.xhtml

The overall goal of the study was to measure the relative contributions of the most predictive correlates of stunting to mean LAZ score difference between stunted and non-stunted children at their 24 months of age. We aimed to generate concrete evidence in helping the policy makers to direct their programs on specific areas of life cycle and to minimize the burden of linear growth faltering. In summary, we have documented that the affect of maternal height and birth weight on linear growth did not decline with age. Our findings indicated the intergenerational transmission of mother's own nutritional status to her offspring. The significance of this association at the policy level would thus ideally aim for the improvement in the nutritional status of a women during her adolescence and pregnancy, which would translate into positive inputs on the birth weight of her offspring and ultimately, on the linear growth of the child. In addition, necessary supports should be provided to ensure sufficient energy intake during early childhood and infancy.

Acknowledgement: The authors would like to thank all the participants and their parents for sharing their time and providing consent, information and biological samples necessary for the successful completion of the study.

Contributions: SD, SEA and TA conceived the study and procured funds. MM developed the data collections tools. MAA managed the data set and provided technical support. SD analyzed the data, developed the tables/graphs and wrote the initial draft of the manuscript. MAA, MM, SEA and TA critically reviewed the manuscript and gave intellectual inputs. All authors contributed to the final version of the paper.

Funding: This paper is made possible by the generous support of the American people through the United States Agency for International Development (USAID) under cooperative agreement no. AID-388-A-17-00006. The views expressed herein are responsibility of the RDM Activity and do not necessarily reflect the views of the U.S. Government or USAID or icddr,b, the implementing agency. The MAL-ED

For peer review only

For peer review only - http://bmjopen.bmj.com/site/about/guidelines.xhtml

(Etiology, Risk Factors, and Interactions of Enteric Infections and Malnutrition and the Consequences for Child Health) birth cohort study was funded by University of Virginia (UVA) with support from MAL-ED Network Investigators in the Foundation of National Institute of Health (FNIH), Fogarty International Centre (FIC) with overall support from the Bill & Melinda Gates Foundation (BMGF). They also acknowledge the contribution of icddr,b's core donors including Government of the People's Republic of Bangladesh, Global Affairs Canada (GAC), Canada; Swedish International Development Cooperation Agency (SIDA) and the Department for International Development, UKAid for their continuous support and commitment to the icddr,b's research efforts.

Competing interests: The authors do not have any competing interests to declare.

Patient consent: Informed written consent was obtained from the mother or primary caregiver.

Data sharing statement: The data sets generated and/or analyzed during the current study is not made publicly available. However, data inquires or further suggestions for analyses can be made to the corresponding author.

REFERENCES

1. Pradhan M, Sahn DE, Younger SD. Decomposing world health inequality. *Journal of health economics* 2003;22(2):271-93.
2. Black RE, Allen LH, Bhutta ZA, et al. Maternal and child undernutrition: global and regional exposures and health consequences. *The lancet* 2008;371(9608):243-60.
3. Victora CG, Adair L, Fall C, et al. Maternal and child undernutrition: consequences for adult health and human capital. *The lancet* 2008;371(9609):340-57.
4. Grantham-McGregor S, Cheung YB, Cueto S, et al. Developmental potential in the first 5 years for children in developing countries. *The lancet* 2007;369(9555):60-70.
5. Fanzo J, Hawkes C, Udomkesmalee E, et al. 2018 Global Nutrition Report: Shining a light to spur action on nutrition. 2018
6. Adekanmbi VT, Uthman OA, Mudasiru OM. Exploring variations in childhood stunting in Nigeria using league table, control chart and spatial analysis. *BMC Public Health* 2013;13(1):361.
7. NIPORT M. Bangladesh Demographic and Health Survey BDHS 2014: Key Indicators. *Dhaka, Bangladesh and Calverton, Maryland, USA: National Institute of Population Research and Training (NIPORT), Mitra and Associates, and ICF International* 2014
8. National Institute of Population Research and Training (NIPORT) ICfDDR, Bangladesh (icddr,b), MEASURE Evaluation. Bangladesh Urban Health Survey 2013 Final Report.

For peer review only - http://bmjopen.bmj.com/site/about/guidelines.xhtml

9. Waterlow J. Introduction. Causes and mechanisms of linear growth retardation (stunting). *Eur J Clin Nutr* 1994;48(Suppl 1):S1-S4.
10. Hoddinott J, Alderman H, Behrman JR, et al. The economic rationale for investing in stunting reduction. *Maternal & Child Nutrition* 2013;9(S2):69-82.
11. Stewart CP, Iannotti L, Dewey KG, et al. Contextualising complementary feeding in a broader framework for stunting prevention. *Maternal & child nutrition* 2013;9:27-45.
12. Hoddinott J, Alderman H, Behrman JR, et al. The economic rationale for investing in stunting reduction. *Maternal & Child Nutrition* 2013;9:69-82.
13. Ahmed T, Mahfuz M, Islam MM, et al. The MAL-ED cohort study in Mirpur, Bangladesh. *Clinical Infectious Diseases* 2014;59(suppl_4):S280-S86.
14. Lohman TG, Roche AF, Martorell R. Anthropometric standardization reference manual: Human kinetics books Champaign 1988.
15. WHO M. Growth, Reference, Study, Group. *WHO Child Growth Standards: Length/height-for-age, weight-for-age, weight-for-length, weight-for-height and body mass index-for-age: Methods and development Geneva: World Health Organization* 2006
16. Caulfield LE, Bose A, Chandyo RK, et al. Infant feeding practices, dietary adequacy, and micronutrient status measures in the MAL-ED study. *Clinical Infectious Diseases* 2014;59(suppl_4):S248-S54.
17. Islam MM, Sanin KI, Mahfuz M, et al. Risk factors of stunting among children living in an urban slum of Bangladesh: findings of a prospective cohort study. *BMC public health* 2018;18(1):197.
18. Daelmans B, Dewey K, Arimond M. New and updated indicators for assessing infant and young child feeding. *Food and nutrition bulletin* 2009;30(2_suppl2):S256-S62.
19. Psaki SR, Seidman JC, Miller M, et al. Measuring socioeconomic status in multicountry studies: results from the eight-country MAL-ED study. *Population health metrics* 2014;12(1):8.
20. Organization WH. Iron deficiency anaemia: assessment, prevention and control: a guide for programme managers. 2001
21. Grant FK, Suchdev PS, Flores-Ayala R, et al. Correcting for Inflammation Changes Estimates of Iron Deficiency among Rural Kenyan Preschool Children–. *The Journal of nutrition* 2011;142(1):105-11.
22. Richard SA, Barrett LJ, Guerrant RL, et al. Disease surveillance methods used in the 8-site MAL-ED cohort study. *Clinical Infectious Diseases* 2014;59(suppl_4):S220-S24.
23. Baqui AH, Black RE, Yunus M, et al. Methodological issues in diarrhoeal diseases epidemiology: definition of diarrhoeal episodes. *International journal of epidemiology* 1991;20(4):1057-63.
24. Morris SS, Cousens SN, Lanata CF, et al. Diarrhoea—defining the episode. *International Journal of Epidemiology* 1994;23(3):617-23.
25. Lanata CF, Rudan I, Boschi-Pinto C, et al. Methodological and quality issues in epidemiological studies of acute lower respiratory infections in children in developing countries. *International journal of epidemiology* 2004;33(6):1362-72.
26. Pio A. Standard case management of pneumonia in children in developing countries: the cornerstone of the acute respiratory infection programme. *Bulletin of the World Health Organization* 2003;81(4):298-300.
27. Diggle P, Liang K, Zeger S. Analysis of Longitudinal Data. Clarendon, Press: Oxford, 1994.
28. Jann B. The Blinder-Oaxaca decomposition for linear regression models. *The Stata Journal* 2008;8(4):453-79.
29. Carey VJ, Lumley T, Ripley B. gee: Generalized Estimation Equation solver, R package version 4.13-18, 2015.
30. Hlavac M. oaxaca: Blinder-Oaxaca Decomposition in R. 2014
31. Ízaltin E, Hill K, Subramanian S. Association of maternal stature with offspring mortality, underweight, and stunting in low-to middle-income countries. *Jama* 2010;303(15):1507-16.

For peer review only - http://bmjopen.bmj.com/site/about/guidelines.xhtml

32. Investigators M-EN. Childhood stunting in relation to the pre-and postnatal environment during the first 2 years of life: The MAL-ED longitudinal birth cohort study. *PLoS medicine* 2017;14(10):e1002408.
33. Hart N. Famine, maternal nutrition and infant mortality: a re-examination of the Dutch hunger winter. *Population Studies* 1993;47(1):27-46.
34. Shrimpton R, Victora CG, de Onis M, et al. Worldwide timing of growth faltering: implications for nutritional interventions. *Pediatrics* 2001;107(5):e75-e75.
35. Mukhopadhyay K, Mahajan R, Louis D, et al. Longitudinal growth of very low birth weight neonates during first year of life and risk factors for malnutrition in a developing country. *Acta Paediatrica* 2013;102(3):278-81.
36. Christian P, Lee SE, Donahue Angel M, et al. Risk of childhood undernutrition related to small-for-gestational age and preterm birth in low-and middle-income countries. *International journal of epidemiology* 2013;42(5):1340-55.
37. Assis AMdO, Prado MdS, Barreto ML, et al. Childhood stunting in Northeast Brazil: the role of Schistosoma mansoni infection and inadequate dietary intake. *European Journal of Clinical Nutrition* 2004;58(7):1022.
38. Mishra V, Retherford RD. Does biofuel smoke contribute to anaemia and stunting in early childhood? *International Journal of Epidemiology* 2006;36(1):117-29.
39. Hautvast J, Van der Heijden L, Luneta A, et al. Food consumption of young stunted and non-stunted children in rural Zambia. *European journal of clinical nutrition* 1999;53(1):50.
40. Wamani H, Åstrøm AN, Peterson S, et al. Boys are more stunted than girls in sub-Saharan Africa: a meta-analysis of 16 demographic and health surveys. *BMC pediatrics* 2007;7(1):17.
41. Cronk L. Low Socioeconomic Status and Female-Biased Parental Investment: The Mukogodo Example. *American Anthropologist* 1989;91(2):414-29.
42. Leslie J, Essama SB, Ciemins E. Female nutritional status across the life-span in sub-Saharan Africa. 2. Causes and consequences. *Food and Nutrition Bulletin* 1997;18(1):1-12.
43. Raj A, McDougal LP, Silverman JG. Gendered effects of siblings on child malnutrition in South Asia: cross-sectional analysis of demographic and health surveys from Bangladesh, India, and Nepal. *Maternal and child health journal* 2015;19(1):217-26.
44. Haider MR, Rahman MM, Islam F, et al. Association of low birthweight and indoor air pollution: Biomass fuel use in Bangladesh. *Journal of Health and Pollution* 2016;6(11):18-25.
45. Oliveira BFAd, Ignotti E, Hacon SS. A systematic review of the physical and chemical characteristics of pollutants from biomass burning and combustion of fossil fuels and health effects in Brazil. *Cadernos de saude publica* 2011;27(9):1678-98.
46. Chen L, Yang W, Jennison BL, et al. Air pollution and birth weight in northern Nevada, 1991-1999. *Inhalation toxicology* 2002;14(2):141-57.

TABLES

Table 1: Maternal-, child-, household-level characteristics and morbidity status of the cohort

n=211 (at 24 month)	All (n=211)	Stunted<br><br>(n=101)	Not stunted<br><br>(n=110)	

For peer review only - http://bmjopen.bmj.com/site/about/guidelines.xhtml

		n (%)			p-value
Maternal-, child- and household-level characteristics					
Sex	Male	108 (51.2)	57 (56.4)	51 (46.4)	0.1
Exclusive breast feeding*	Yes	23 (10.9)	12 (11.9)	11 (10.0)	0.66
Household head's sex	Male	195 (92.4)	95 (94.1)	100 (90.9)	0.83
Mother's age at birth (years)	<18y	3 (1.4)	0 (0.0)	3 (2.7)	0.2
	18-30y	178 (84.4)	84 (83.2)	94 (85.5)	
	>30y	30 (14.2)	17 (16.8)	13 (11.8)	
Mother's age at first pregnancy	<18y	84 (39.8)	34 (33.7)	50 (45.5)	0.08
Maternal education	No education	41 (19.4)	22 (21.8)	19 (17.3)	0.71
	<5y	93 (44.1)	43 (42.6)	50 (45.5)	
	>5y	77 (36.5)	36 (35.6)	41 (37.3)	
Separate room for a kitchen	Yes	17 (8.1)	4 (4.0)	13 (11.8)	0.04
Monthly family income	<100 USD	94 (44.5)	52 (51.5)	42 (38.2)	0.05
Households have chicken or ducks	Yes	13 (6.2)	5 (5.0)	8 (7.3)	0.48
Treat water to make it safe	Yes	135 (64.0)	56 (55.4)	78 (70.9)	0.02
		Mean (SD)			
Exclusive breast feeding days (ebf days)		102.00 (57.7)	105.00 (56.9)	99.6 (58.5)	<0.00
WAMI score**		0.53 (0.13)	0.50 (0.13)	0.55 (0.13)	0.17
People per room		3.57 (1.18)	3.83 (1.32)	3.33 (0.98)	<0.00

For peer review only

Birth weight (kg)	2.82 (0.42)	2.70(0.39)	2.92(0.42)	<0.00
Maternal height (cm)	149.03 (5.05)	147.34 (4.85)	150.54 (4.76)	<0.00
Morbidity episodes/child-year				
Diarrhea	3.63	4.03	3.43	0.08
ALRI	0.47	0.54	0.46	0.98
Fever	8.06	8.78	7.87	0.74
Cough	9.69	9.64	9.83	0.02

* Excluding breast feeding: Exclusive breastfeeding is defined as no other food or drink, not even water, except breast milk (including milk expressed or from a wet nurse) for 6 months of life, but allows the infant to receive ORS, drops and syrups (vitamins, minerals and medicines)

**The WAMI score (ranging from 0 to 1) is a measure of household socioeconomic status including access to improved Water, sanitation and hygiene (WASH), Assets, Maternal education, and Income.

Table 2: Determinants of stunting among children: results of Generalized Estimating Equation modeling (*Dependent variable: Stunted no vs.yes)*

	Unadjusted OR (95% CI	p-value	Adjusted OR (95% CI	p-value
**Energy intake*(kcal)**	**1.001 (1.001 -1.001)**	**0.01**	**1.001 (1.001-1.002)**	**<0.00**
Ebf days	1.002 (1.001 - 1.003)	<0.00	1.002 (1.002-1.002)	0.81
Minimum acceptable diet	0.74 (0.64 - 0.85)	<0.00	0.89 (0.72 - 1.09)	0.25
Cough episodes (per child-year)	0.90 (0.82 - 0.98)	<0.00	0.97 (0.86 - 1.10)	0.67

For peer review only - http://bmjopen.bmj.com/site/about/guidelines.xhtml

Anemia**	1.45 (1.26 - 1.69)	<0.00	1.09 (0.90 - 1.32)	0.40
**Gender**	**0.70 (0.61 - 0.80)**	**<0.00**	**0.47 (0.38 - 0.58)**	**<0.00**
**Maternal height (cm)***	**0.89 (0.88 - 0.91)**	**<0.00**	**0.87 (0.86 - 0.89)**	**<0.00**
**Separate room for a kitchen**	**0.37 (0.28 - 0.50)**	**<0.00**	**0.21 (0.12 - 0.38)**	**<0.00**
WAMI	0.06 (0.04 - 0.11)	<0.00	0.39 (0.15 - 1.03)	0.06
**People per room***	**1.34 (1.26 - 1.42)**	**<0.00**	**1.48 (1.35 - 1.62)**	**<0.00**
**Birth weight (kg)***	**0.18 (0.15 - 0.22)**	**<0.00**	**0.11 (0.09 - 0.15)**	**<0.00**
Serum Zinc (mmol/L)	0.94 (0.91 - 0.98)	<0.00	0.99 (0.95 - 1.04)	0.79

434 OR= Odds ratio; CI= Confidence intervals

435 *per one unit change

436 ** Anemia is defined as a hemoglobin concentration <110 g/L

437 **Table 3:** Relative contribution of the selected variables on mean length-for-age Z score difference

438 between stunted and non-stunted children at 24 month

	Endowment			Coefficient			Interaction		
	Coef.	95% CI		Coef.	95% CI		Coef.	95% CI	
		Upper	Lower		Upper	Lower		Upper	Lower
Overall	0.26	0.41	0.10	1.34	1.50	1.19	-0.12	0.03	-0.26

For peer review only - http://bmjopen.bmj.com/site/about/guidelines.xhtml

Energy intake (kcal)	0.00	0.03	-0.03	-0.12	0.22	-0.46	0.00	0.03	-0.03
Gender	0.01	0.02	-0.01	0.02	0.11	-0.07	0.00	0.01	-0.02
Maternal height (cm)	0.10	0.18	0.02	-3.04	0.35	-6.44	-0.07	0.02	-0.15
Separate kitchen	0.00	0.03	-0.03	0.01	0.03	-0.02	0.02	0.06	-0.03
People per room	0.06	0.12	0.00	0.31	0.92	-0.30	-0.04	0.04	-0.12
Birth weight (kg)	0.08	0.17	-0.01	-0.21	0.88	-1.30	-0.02	0.07	-0.11

Figure legends

Figure 1

Title: Patterns of macronutrient intake from 9 to 24 months of age

Legends: Mean macronutrient intake per day (grams), Months of age, Animal protein, Carbohydrate, Fat, Total protein, Stunted, Not stunted

Figure 2

Title: Prevalence of stunting among children who did not take Minimum Acceptable Diet (MAD) and those who took it (MAD: For breastfed children MAD is defined as who had at least the minimum dietary diversity and the minimum meal frequency during the previous day and for non-breastfed children it is defined as who received at least 2 milk feedings and had at least the minimum dietary diversity not including milk feeds and the minimum meal frequency during the previous day)

Legends: Prevalence of stunting (95 % CI), Months of age, Did not take MAD, Took MAD

Supplementary figure 1

Title: Serum zinc, ferritin and hemoglobin levels of stunted and non-stunted children at 7, 15, and 24 months of age

Legends: Zinc (mmol/L), Ferritin (ug /L), Hemoglobin (g/dL), Stunted, No, Yes

Supplementary figure 2

For peer review only

For peer review only - http://bmjopen.bmj.com/site/about/guidelines.xhtml

457 Title: Coefficient plot of relative contributions of the selected variables on mean LAZ score difference
458 between stunted and non-stunted children at 24 months of age

459 Legends: Coefficients of relative contribution, Energy intake, Gender, Maternal height, Separate kitchen,
460 Crowding, Birth weight

461

For peer review only

For peer review only - http://bmjopen.bmj.com/site/about/guidelines.xhtml

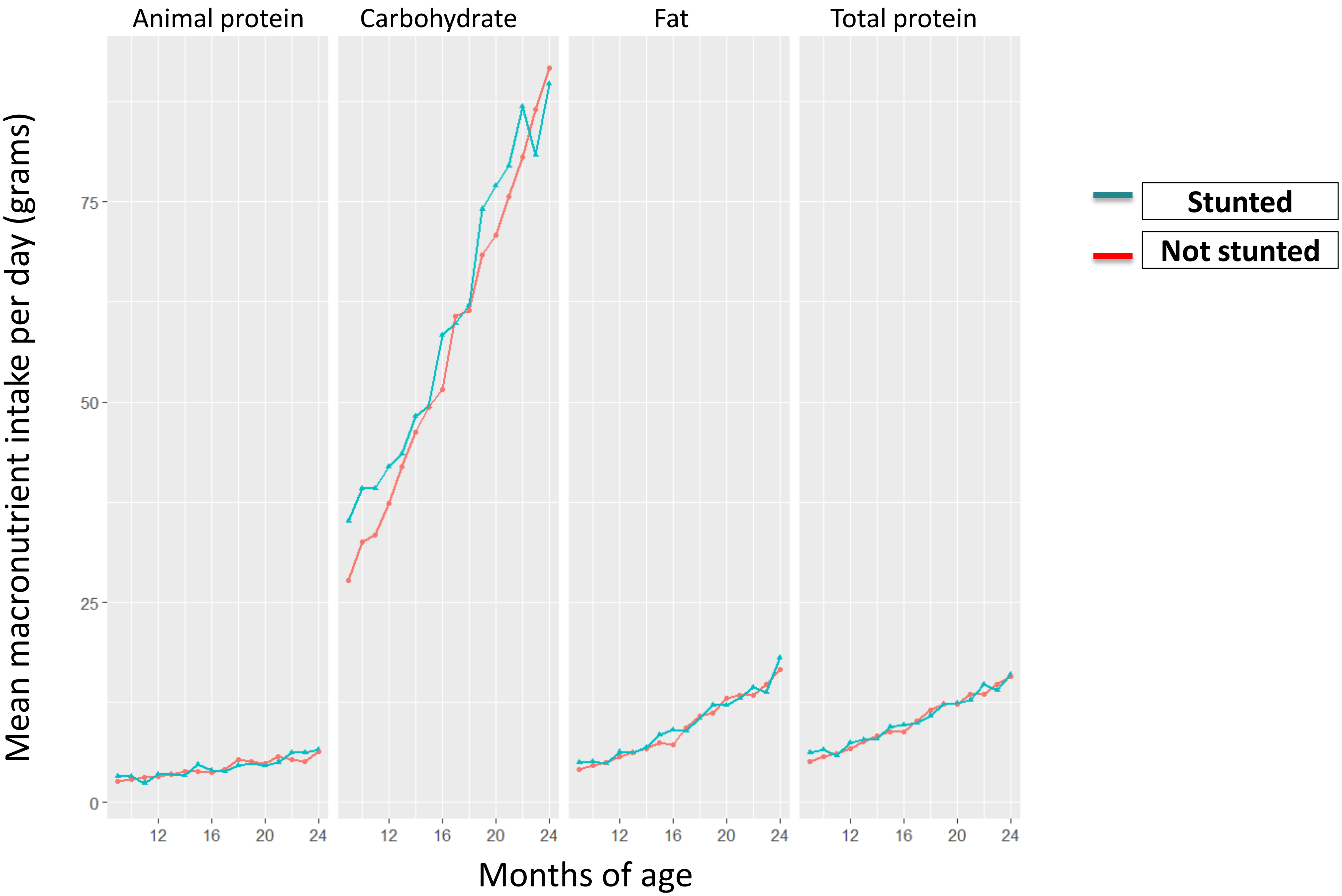
Animal protein
Carbohydrate
Fat
Total protein
Mean macronutrient intake per day (grams)
75
50
25
0
12
16
20
24
Months of age
Stunted
Not stunted

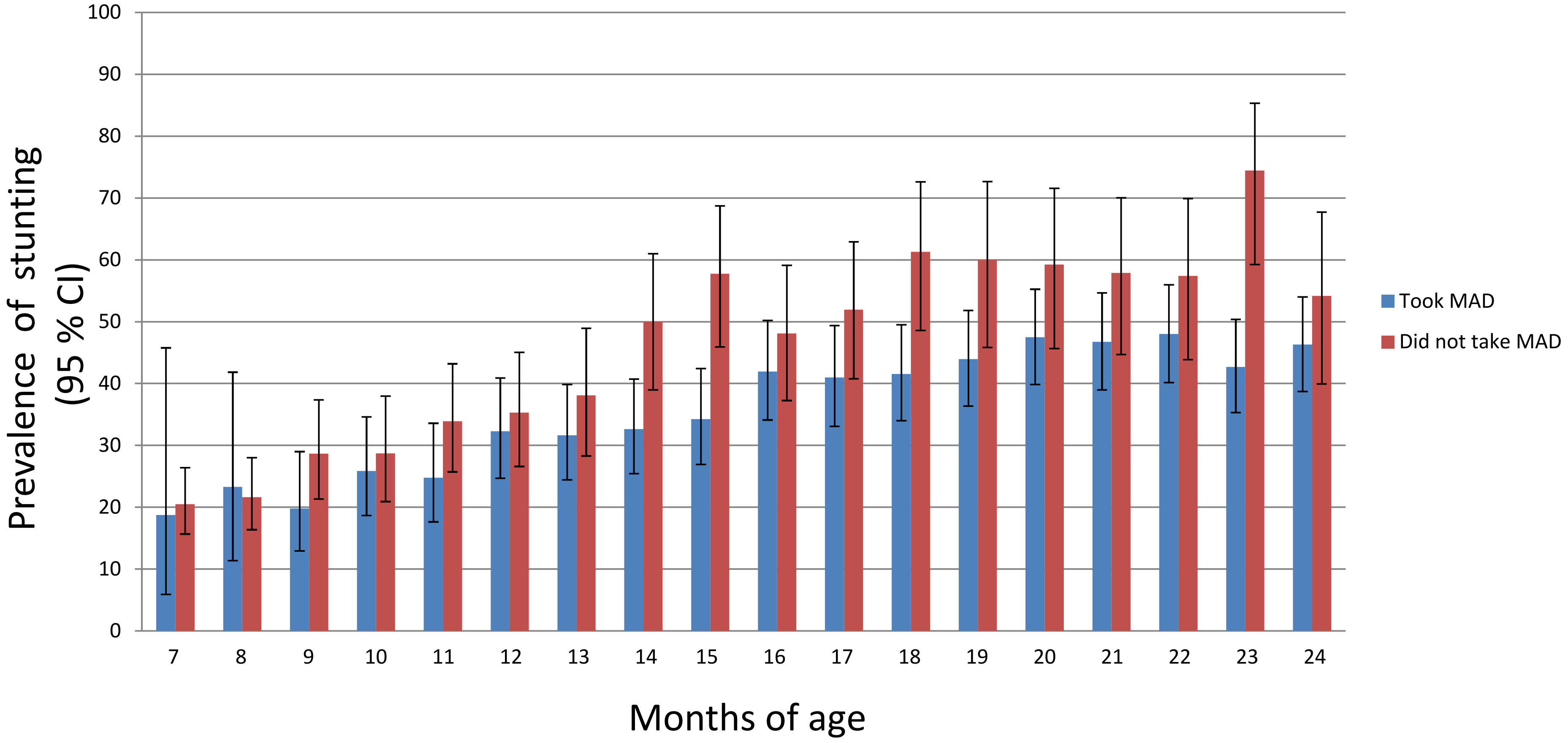
100
90
80
70
60
50
40
30
20
10
0
Prevalence of stunting (95 % CI)
7
8
9
10
11
12
13
14
15
16
17
18
19
20
21
22
23
24
Months of age
Took MAD
Did not take MAD

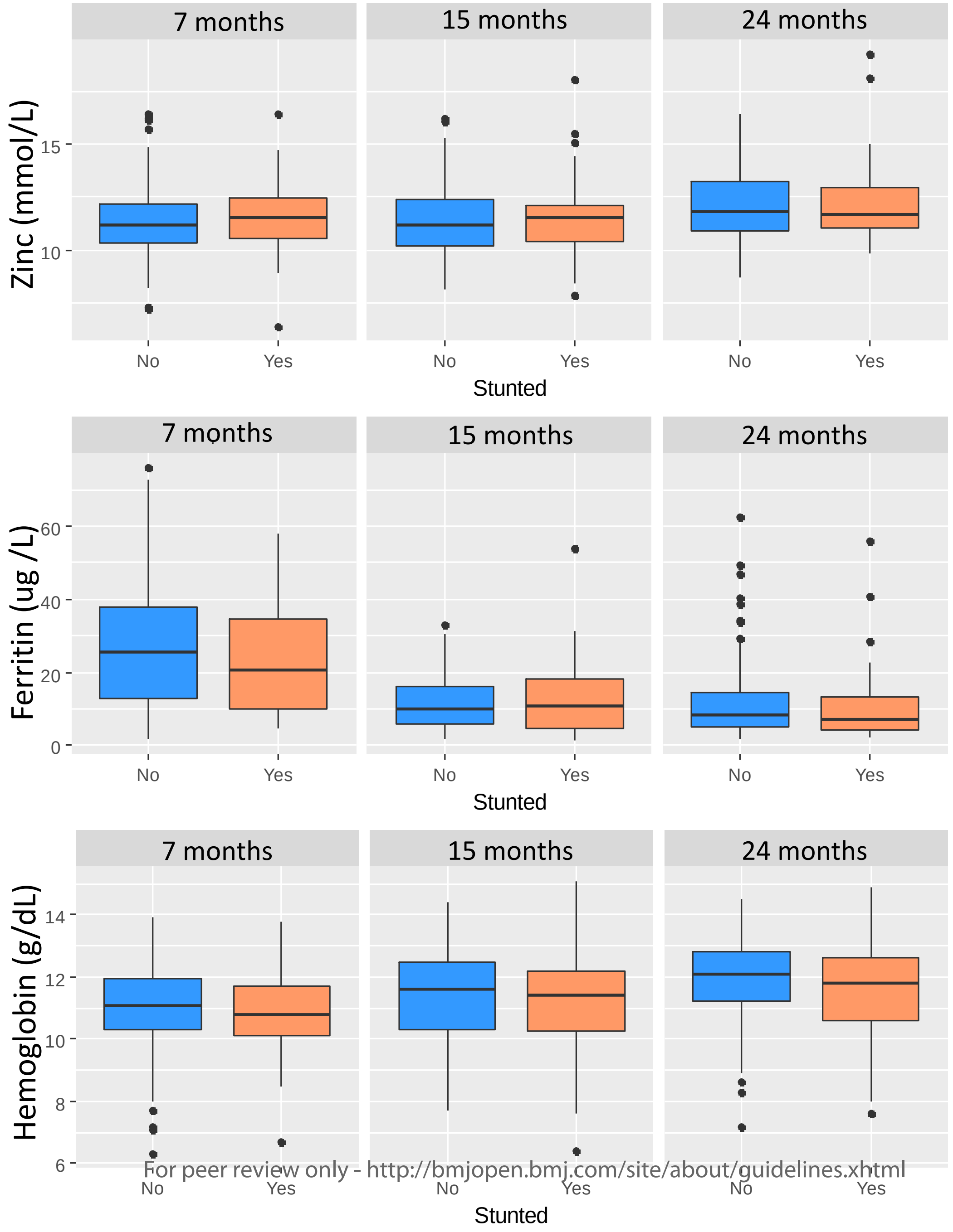

For peer review only - http://bmjopen.bmj.com/site/about/guidelines.xhtml

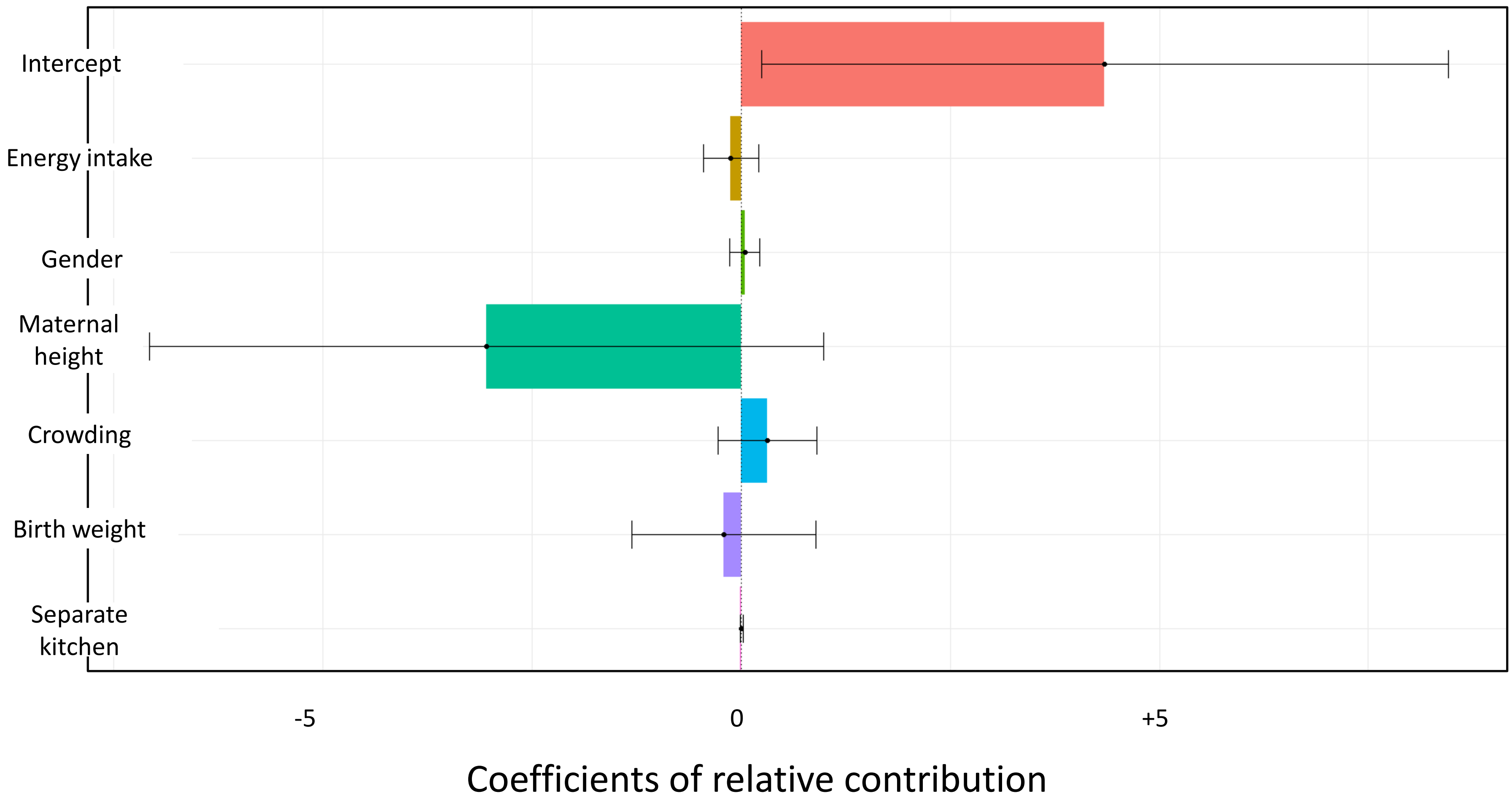
Intercept
Energy intake
Gender
Maternal height
Crowding
Birth weight
Separate kitchen
-5
0
+5
Coefficients of relative contribution

STROBE (Strengthening The Reporting of OBservational Studies in Epidemiology) Checklist

A checklist of items that should be included in reports of observational studies. You must report the page number in your manuscript where you consider each of the items listed in this checklist. If you have not included this information, either revise your manuscript accordingly before submitting or note N/A.

Note: An Explanation and Elaboration article discusses each checklist item and gives methodological background and published examples of transparent reporting. The STROBE checklist is best used in conjunction with this article (freely available on the Web sites of PLoS Medicine at http://www.plosmedicine.org/, Annals of Internal Medicine at http://www.annals.org/, and Epidemiology at http://www.epidem.com/). Information on the STROBE Initiative is available at www.strobe-statement.org.

Section and Item	Item No.	Recommendation	Reported on Page No.
**Title and Abstract**	1	(*a*) Indicate the study's design with a commonly used term in the title or the abstract	1,2
		(*b*) Provide in the abstract an informative and balanced summary of what was done and what was found	1,2
**Introduction**			
Background/Rationale	2	Explain the scientific background and rationale for the investigation being reported	3-4
Objectives	3	State specific objectives, including any prespecified hypotheses	4
**Methods**			
Study Design	4	Present key elements of study design early in the paper	4
Setting	5	Describe the setting, locations, and relevant dates, including periods of recruitment, exposure, follow-up, and data collection	4
Participants	6	(*a*) *Cohort study*—Give the eligibility criteria, and the sources and methods of selection of participants. Describe methods of follow-up	4
		Case-control study—Give the eligibility criteria, and the sources and methods of case ascertainment and control selection. Give the rationale for the choice of cases and controls	n/a
		Cross-sectional study—Give the eligibility criteria, and the sources and methods of selection of participants	n/a
		(*b*) *Cohort study*—For matched studies, give matching criteria and number of exposed and unexposed	n/a
		Case-control study—For matched studies, give matching criteria and the number of controls per case	n/a
Variables	7	Clearly define all outcomes, exposures, predictors, potential confounders, and effect modifiers. Give diagnostic criteria, if applicable	5-6

For peer review only

Section and Item	Item No.	Recommendation	Reported on Page No.
Data Sources/ Measurement	8*	For each variable of interest, give sources of data and details of methods of assessment (measurement). Describe comparability of assessment methods if there is more than one group	5-6
Bias	9	Describe any efforts to address potential sources of bias	7
Study Size	10	Explain how the study size was arrived at	4
Quantitative Variables	11	Explain how quantitative variables were handled in the analyses. If applicable, describe which groupings were chosen and why	7
Statistical Methods	12	(*a*) Describe all statistical methods, including those used to control for confounding	7
		(*b*) Describe any methods used to examine subgroups and interactions	n/a
		(*c*) Explain how missing data were addressed	7
		(*d*) *Cohort study*—If applicable, explain how loss to follow-up was addressed	7
		Case-control study—If applicable, explain how matching of cases and controls was addressed	n/a
		Cross-sectional study—If applicable, describe analytical methods taking account of sampling strategy	n/a
		(*e*) Describe any sensitivity analyses	n/a
**Results**			
Participants	13*	(a) Report numbers of individuals at each stage of study—eg numbers potentially eligible, examined for eligibility, confirmed eligible, included in the study, completing follow-up, and analysed	7
		(b) Give reasons for non-participation at each stage	n/a
		(c) Consider use of a flow diagram	n/a
Descriptive Data	14*	(a) Give characteristics of study participants (eg demographic, clinical, social) and information on exposures and potential confounders	7,8
		(b) Indicate number of participants with missing data for each variable of interest	n/a
		(c) *Cohort study*—Summarise follow-up time (eg, average and total amount)	4
Outcome Data	15*	*Cohort study*—Report numbers of outcome events or summary measures over time	7
		Case-control study—Report numbers in each exposure category, or summary measures of exposure	
		Cross-sectional study—Report numbers of outcome events or summary measures	

For peer review only

Section and Item	Item No.	Recommendation	Reported on Page No.
Main Results	16	(*a*) Give unadjusted estimates and, if applicable, confounder-adjusted estimates and their precision (eg, 95% confidence interval). Make clear which confounders were adjusted for and why they were included	9,17
		(*b*) Report category boundaries when continuous variables were categorized	n/a
		(*c*) If relevant, consider translating estimates of relative risk into absolute risk for a meaningful time period	n/a
Other Analyses	17	Report other analyses done—eg analyses of subgroups and interactions, and sensitivity analyses	n/a
**Discussion**			
Key Results	18	Summarise key results with reference to study objectives	10,11
Limitations	19	Discuss limitations of the study, taking into account sources of potential bias or imprecision. Discuss both direction and magnitude of any potential bias	11
Interpretation	20	Give a cautious overall interpretation of results considering objectives, limitations, multiplicity of analyses, results from similar studies, and other relevant evidence	10,11
Generalisability	21	Discuss the generalisability (external validity) of the study results	10,11
**Other Information**			
Funding	22	Give the source of funding and the role of the funders for the present study and, if applicable, for the original study on which the present article is based	12

*Give information separately for cases and controls in case-control studies and, if applicable, for exposed and unexposed groups in cohort and cross-sectional studies.

Once you have completed this checklist, please save a copy and upload it as part of your submission. DO NOT include this checklist as part of the main manuscript document. It must be uploaded as a separate file.

For peer review only

BMJ Open

Relative contributions of the correlates of stunting in explaining the mean length-for-age z-score difference between 24-month-old stunted and non-stunted children living in a slum of Dhaka, Bangladesh: Results from a decomposition analysis

Journal:	*BMJ Open*
Manuscript ID	bmjopen-2018-025439.R2
Article Type:	Research
Date Submitted by the Author:	07-May-2019
Complete List of Authors:	Das, Subhasish; International Centre for Diarrhoeal Disease Research, Bangladesh (icddr,b), Nutrition and Clinical Services Division; Employer<br>Alam, Md Ashraful; International Centre for Diarrhoeal Disease Research Bangladesh, Nutrition and Clinical Services Division<br>Mahfuz, Mustafa; International Centre for Diarrhoeal Disease Research, Nutrition and Clinical Services Division<br>Arifeen, Shams E.; ICDDR B, Maternal and Child Health Division (MCHD)<br>Ahmed, Tahmeed; International Centre for Diarrhoeal Disease Research, Bangladesh (icddr,b), Nutrition and Clinical Services Division (NCSD)
**Primary Subject Heading**:	Public health
Secondary Subject Heading:	Epidemiology, Global health, Nutrition and metabolism, Public health
Keywords:	Relative contribution, Decomposition analysis, Maternal height, Stunting, Length-for-age z-score

SCHOLARONE™
Manuscripts

TITLE: Relative contributions of the correlates of stunting in explaining the mean length-for-age z-score difference between 24-month-old stunted and non-stunted children living in a slum of Dhaka, Bangladesh: Results from a decomposition analysis

Corresponding author:

Subhasish Das, Nutrition and Clinical Services Division, International Centre for Diarrhoeal Disease Research Bangladesh (icddr,b), Dhaka 1212, Bangladesh. Email: subhasish.das@icddrb.org. Telephone number: +8801617099766

Co-authors:

Md Ashraful Alam, Nutrition and Clinical Services Division, icddr,b, Dhaka, Bangladesh

Mustafa Mahfuz, Nutrition and Clinical Services Division, icddr,b, Dhaka, Bangladesh

Shams El Arifeen, Maternal and Child Health Division, icddr,b, Dhaka, Bangladesh

Tahmeed Ahmed, Nutrition and Clinical Services Division, icddr,b, Dhaka, Bangladesh

Authors' email addresses: subhasish.das@icddrb.org, mashraful@icddrb.org, mustafa@icdddrb.org, shams@icddrb.org, tahmeed@icddrb.org

Body text word count: 3471

Keywords: Stunting; Length-for-age z-score; Relative contribution; Decomposition analysis; Maternal height.

ABSTRACT

For peer review only - http://bmjopen.bmj.com/site/about/guidelines.xhtml

Objective: Using MAL-ED Bangladesh birth cohort data we sought to measure the relative contributions of the most predictive correlates of stunting to mean length-for-age z (LAZ) score difference between stunted and non-stunted children at 24 months of age.

Setting: Dhaka, Bangladesh

Participants: 211 slum dwelling children enrolled within 17 days of their birth.

Variables and method: The explanatory variables were identified from following groups: maternal characteristics, birth characteristics, macronutrient intake, socio-economic status, morbidity and serum micronutrient level. At step 1, predictive correlates of stunting were identified longitudinally (from 9 to 24 months of age) using generalized estimating equations (GEE) model. Then, the relative contributions of the most predictive correlates of stunting to mean LAZ score difference between stunted and non-stunted children at 24 months of age was measured using Blinder-Oaxaca decomposition analysis.

Results: The GEE multivariable model identified maternal height, birth weight, people per room, gender, having separate room for kitchen and energy intake as the most predictive correlates of stunting. At 24 months, mean LAZ score difference between stunted and non-stunted children was 1.48. The variable by variable decomposition of the LAZ gap identified maternal height (coefficient: -3.04 ; 95% CI: 0.35, -6.44), birth weight (coefficient: -0.21; 95% CI: 0.88, -1.30), people per room (coefficient: 0.31; 95% CI: 0.92, -0.30) and energy intake (coefficient: -0.12; 95% CI: 0.22, -0.46) as the top most factors responsible for the mean LAZ score difference between stunted and non-stunted children at 24 months of age.

Conclusions: The relative contributions of maternal height and birth weight to LAZ gap signifies that improvement in nutritional status of a women during her adolescence and pregnancy would have an impact on birth weight of her offspring, and ultimately, on linear growth of the child.

For peer review only - http://bmjopen.bmj.com/site/about/guidelines.xhtml

Strengths and limitations of this study

- This study used the threefold Blinder-Oaxaca decomposition analysis to decompose the relative contributions of most predictive correlates of stunting to mean LAZ score difference of stunted and non-stunted children into three components: endowments, coefficients, and the interaction of the two.
- The explanatory variables used for the decomposition analysis were identified after analyzing the longitudinal birth cohort data collected from the same children.
- At 24 months, 20% of the enrolled children were lost to follow-up which could lead to possible bias.
- The findings of this study might not be applicable to the non-slum setting, as this study was conducted in a slum area of Dhaka, Bangladesh.

INTRODUCTION

Stunting (length-for-age more than two standard deviations below the WHO Child Growth Standards median) is an indicator of chronic undernutrition and is one of the best measures of child health inequality.[1] It is one of the most prevalent forms of malnutrition which is known to be associated with higher mortality and cognitive impairment of affected children.[1-4] Worldwide, 150.8 million under 5 children are stunted and in Asia, the prevalence is 23.2%.[5] Variation in rates of stunting exists not only across continents but also within local geographies.[6] The prevalence of stunting in Bangladeshi slum areas is much higher than the national level average (50 vs. 36 %, respectively). [7 8]

Children from lowest wealth quintiles who have a normal anthropometric status at birth may experience an intense period of growth retardation.[9] During the subsequent life-cycle stages this growth

For peer review only - http://bmjopen.bmj.com/site/about/guidelines.xhtml

retardation continues to exert negative effects such as socio-emotional impairment in pre-school age, poor school attainment in early childhood, and higher risk of chronic disease in adulthood.[10] WHO conceptual framework on childhood stunting reported maternal factors, home environment, inadequate complementary feeding, breast feeding and infection as the most proximal factors responsible for stunting.[11] But, relative contributions of these correlates to length-for-age z (LAZ) score difference between stunted and non-stunted children were not measured. In high burden countries the benefit-cost ratio of investments to reduce stunting ranges from 3.8 to 47.9, whereas the ratio is 18.4 in Bangladesh.[12] To that end, since Bangladesh is a resource constrained country, identifying the top contributors of stunting would help the policy-makers to direct concerted stunting prevention programs in a cost effective way. Keeping the context in mind, we identified the correlates of stunting among 9-24 months old children using the longitudinal data emanated from a birth cohort study. Then, we measured the relative contributions of the most predictive correlates of stunting in explaining the mean LAZ score difference between stunted and non-stunted children at 24 months of age.

METHODS

Study participants

Data used for this analysis was collected from the MAL-ED (Etiology, Risk Factors, and Interactions of Enteric Infections and Malnutrition and the Consequences for Child Health) Bangladesh site. The MAL-ED birth cohort study was conducted at 8 sites in 3 continents. In Bangladesh the MAL-ED study site was Bauniabadh slum, Mirpur, Dhaka which was described elsewhere.[13] Following a well-defined protocol, field workers visited households and enrolled 265 healthy newborn children within 17 days of their birth between February 2010 and February 2012. Participants were followed till 24 months of age. At 24 months, 211 participants remained which is 80% of the total recruitment. The study was approved by the Institutional Review Board of icddr,b (protocol no. PR-2008-020).

For peer review only

For peer review only - http://bmjopen.bmj.com/site/about/guidelines.xhtml

Data collection

Dependent variable

Following standard anthropometric methodology trained field workers measured the recumbent length of the children to the nearest 1 mm using Seca infantometer (model no.417, Hamburg, Germany) every month until 24 months of age.[14] The LAZ score of each child was determined using the WHO 2006 child growth standards.[15] A child with LAZ < –2 is classified as stunted.[15]

Independent variables

The explanatory variables used for the analysis were drawn from the following groups: birth characteristics, maternal characteristics, nutrient intake, socio-economic status, morbidity and serum micronutrient. Data on birth characteristics (enrollment weight and length) and maternal height were collected within 72 hours of recruitment. Children, wearing minimum cloths, were weighed using a digital scale with 10 g precision (Seca, model no.345, Hamburg, Germany). The enrollment weight was used as the surrogate measure for birth weight. Standard wooden height measuring boards were used for measuring maternal height. For assessing the feeding habit and energy intake of the children, 24-hour food frequency data was collected monthly from 9th month onwards using a 24-hour multiple-pass dietary recall approach.[16] The 24-hour dietary recall interviews were conducted on non-consecutive days and out of every four recalls, one interview was conducted on a weekend. A locally adapted food composition table was used to convert the dietary intake data to energy.[17] Minimum acceptable diet (MAD)- a core indicator of infant and young child feeding practice was used to measure the appropriateness of the complementary feeding practice of the children.[18] MAD meets standards for both minimum dietary diversity and minimum meal frequency. Socio-economic data was collected at 6, 12, 18 and 24 months. WAMI index (**W**ater, sanitation, hygiene, **A**sset, **M**aternal education and **I**ncome

For peer review only - http://bmjopen.bmj.com/site/about/guidelines.xhtml

index; ranging from 0 to 1) -a socio-economic status index which includes access to improved water and sanitation, eight selected assets, maternal education, and household income was used as a representative of socio-economic status of the households.[19] A higher WAMI index indicates a better socio-economic status. To measure the household level crowding a continuous variable named "people per room" was generated by dividing the total number of people usually slept in the household by the number of rooms of that household used for sleeping. Blood samples for measuring serum zinc, ferritin and hemoglobin status were collected at 7, 15 and 24 months of age of a child. For hemoglobin, capillary blood was collected and measured with the Hemocue device (Hb 201, Ängelholm, Sweden). Plasma zinc and ferritin levels were measured using atomic absorption spectometry (AAS) and chemiluminescence immunoassay (CLIA), respectively. Anemia is defined as a hemoglobin concentration <110 g/L.[20] Plasma ferritin and zinc concentration values were adjusted for the effect of subclinical inflammation using both C-reactive protein (CRP) and α-1-acid glycoprotein (AGP).[21]

Trained field workers visited the participant's household twice a week and used a surveillance assessment form (SAF) to document the breast feeding and illness status of the child. Household visits were generally made on every 3–4 days. During every such visits, field workers asked the caregiver whether the children experienced any of the symptoms listed in SAF on each day since the last visit.[22] To document the breastfeeding status, the staff asked the mother about the liquids the child consumed during the past 24 hours. If the response followed the WHO definition of exclusive breast feeding (no other food or drink, not even water, except breast milk (including milk expressed, ORS, drops and vitamins, minerals and medicines syrups)), then the child was considered as exclusively breast fed. Diarrhea is defined as having ≥3 loose stools in a 24-hour period or at least 1 loose stool with blood reported by the mother. [23] A diarrheal episode is defined as being separated from another episode by at least 2 or more diarrhea-free days.[23-25] Acute lower respiratory infection (ALRI) is identified based on the presence of cough and/or shortness of breath plus high respiratory rate.[25] The average of two

For peer review only - http://bmjopen.bmj.com/site/about/guidelines.xhtml

respiratory rates was used, and was considered high based on the age of the child (<2 months of age: ≥60 breaths/minute; 2 to <12 months of age: ≥50 breaths/minute; ≥12 months of age: ≥40 breaths/minute).[26] ALRI episodes are separated by at least 15 ALRI-free days. [25] Fever is defined as an axillary temperature >37.5°C. [22]

Statistics

We reported the maternal-, child-, and household-level characteristics of the study participants using mean and standard deviation for continuous variables and frequency as percentages for categorical variables. Student's t-test, Chi-square test or the Fisher's exact test was used to compare the above mentioned characteristics of the stunted children to their non-stunted counterparts at 24 months of age.

At stage 1, we analyzed the longitudinal birth cohort data to identify the most predictive correlates of stunting. Serum micronutrient data was collected at 7, 15 and 24 months of age of the children. We carried forward 7 months data up to 14 months and 15 months data up to 23 months. Dietary intake data collection started at 9 months of age. Hence, to ensure the availability of maximum variables at every time point and temporality of the relation between outcome and explanatory variables, the longitudinal data analysis was done using the data collected from 9 to 24 months of age. Instead of exclusive breast feeding (ebf) status (yes vs. no), exclusive breast feeding days (ebf days) was used as it counts the specific number of days. The association between each explanatory variable and the binary outcome variable (stunting: no vs. yes) was examined longitudinally using population specific generalized estimation equations (GEE).[27] The generalized estimating equations (GEE) method is an extension of the quasi-likelihood approach that permits specification of a working correlation matrix for the within-subject correlation of repeated responses collected from the same participants over time. As a result, more efficient and unbiased regression parameters are produced. Initially, bivariate analyses

For peer review only - http://bmjopen.bmj.com/site/about/guidelines.xhtml

were performed to identify the unadjusted effect of each explanatory variable on the outcome variable. The explanatory variables showing statistically significant association (p value <0.05) with the outcome variable in the bivariate analyses were selected for multivariable modeling. The variance inflation factor (VIF) was calculated to detect multicollinearity and variables producing a VIF value > 5 were excluded from the analysis. Quasi-likelihood information criterion (QIC) was used to select the correct covariance structure. The multivariable model with unstructured covariance matrix produced the smallest QIC value. Hence, we report the results of the multivariable model that was constructed using unstructured covariance matrix with robust variance estimates. We determined the strength of association by estimating the odds ratios and their 95% confidence intervals. Statistical significance was determined at a probability value less than 0.05 and the variables showing statistically significant association with stunting in the multivariable GEE model were selected for the decomposition analysis.

At stage 2, threefold Blinder-Oaxaca decomposition analysis was done to measure the relative contribution of the identified variables to mean length-for-age z-score difference between stunted and non-stunted children at 24 months of age. The threefold decomposition analysis decomposes the gap or difference of the group-specific means into three components- a gap due to differences in the level or magnitude of determinants or endowments, a gap due to the differences in the effects of the determinants or coefficients, and a gap due to the interactions between endowments and coefficients.[28] During decomposition analysis bootstrapped standard errors were calculated based on 1,000 replicates. R version 3.4.2 was used for data analysis and GEE and Oaxaca packages were employed.[29 30]

Patient and Public Involvement (PPI) statement

No patient or public were involved with the development of research question, designing the study, recruitment of participants, interpretation of the results, and will be involved during disseminating the findings of the paper.

For peer review only - http://bmjopen.bmj.com/site/about/guidelines.xhtml

RESULTS

At month 1, the cohort exhibited a 19% prevalence of stunting. The prevalence showed a similar trend till month 6 (18.4 %). After that, this trend went up to 33.6%, 46.8% and 47.9% at 12, 18 and 24th months, respectively.

The socio-demographic characteristics of the participating households are described in table 1. Till 24 months, the study was able to follow a cohort of 211 children. At 24 months, the overall male to female ratio was 1.04, which was 1.3 in the stunted cohort. Only 10.9% of children were exclusively breastfed, the prevalence of which did not change over the stunting status. Most of the households were headed by a male family member. One-fifth of the mothers were illiterate and nearly half of them had an education of less than 5 years. Nearly half of the households had a monthly income of less than 100 USD, the percentage of which was higher in the stunted group than the non-stunted group. Mean birth weight of the stunted children was lower than their non-stunted counterparts. The mothers of the stunted children also had a lower height than those of the non-stunted children.

Morbidity data displayed in table 1 show that per year stunted children suffered from more than 4 diarrheal episodes which was higher than the overall annual rate of 3.63 episodes. Similar trends were seen for ALRI and fever episodes. However, the stunted - non stunted differences in the diarrheal, ALRI and fever episodes are not statistically significant ($p > 0.05$). Conversely, the non-stunted group had a higher average of cough episodes ($p = 0.02$) than their stunted counterparts.

Figure 1 represents the pattern of macronutrient intake from 9 to 24 months. Over the period, the trend of nutrient intake did not differ much between the stunted and non-stunted groups. For both the groups, carbohydrate was the main source of nutrition.

For peer review only - http://bmjopen.bmj.com/site/about/guidelines.xhtml

Figure 2 presents the comparative picture of prevalence of stunting among the children with and without minimum acceptable diet (MAD). Children without minimally acceptable diets were more stunted than children with minimally acceptable diets, and from 9 months onwards the difference in prevalence of stunting between these two groups increased with age. However, except at 15 and 23 months, the confidence intervals of prevalence largely overlap. Hence, for most the months, the prevalence of stunting did not show statistically significant differences between the MAD and non-MAD groups.

Box plots reported in supplementary figure 1 show the comparative picture of serum zinc, ferritin and hemoglobin level of stunted and non-stunted children at 7, 15, and 24 months. The figure reveals that there were no notable differences in serum zinc, ferritin and hemoglobin levels between stunted and not stunted children at 7, 15, and 24 months.

Table 2 presents the results of generalized estimating equation modeling. Thorough literature review was done to identify the variables of interest and bivariate GEE modeling was carried out longitudinally by taking into account all the variables, one at a time. However, table 2 shows only those variables found to have a statistically significant association (p value <0.05) with stunting. From nutrient intake group the amount of energy intake was selected because separate food groups (carbohydrate, total protein, animal protein and fat) showed a very high correlation with each other. The multivariable model showed significant (p value < 0.05) association of energy intake, gender, maternal height, having a separate room for kitchen, people per room and birth weight with stunting.

Table 3 and supplementary figure 2 illustrate the results of Blinder-Oaxaca decomposition analysis. At month 24, the mean LAZ score of non-stunted group was -1.33 and in the stunted group it was -2.81, yielding a LAZ gap of 1.48. The stunted-non stunted LAZ gap had subsequently been decomposed into three components- a gap due to differences in the level or magnitude of determinants (endowments), a

For peer review only

For peer review only - http://bmjopen.bmj.com/site/about/guidelines.xhtml

gap due to the differences in the effects of the determinants (coefficients) and a gap due to the interactions between endowments and coefficients. The results of the threefold decomposition suggest that, of the 1.48 difference, approximately 0.26 (95% CI: 0.41, 0.10) was due to the group differences in endowments, 1.34 (95% CI: 1.50, 1.19) to differences in coefficients, and the remaining -0.12 (95% CI: 0.03, -0.26) was for the interaction of the two. The 'endowment' part of the result reflects the average increase in stunted children's LAZ if they had the same level of characteristics as non-stunted children. This also indicates that the differences in the magnitude of explanatory variables account for about 17.6% of the LAZ gap among stunted and non-stunted children. The coefficient part shows that if the effect of the non-stunted children's characteristics was applied to the stunted children, the changes in the LAZ of the stunted cohort would be 1.34. The interaction component indicates that the simultaneous effect of endowment and coefficient was -0.12. The findings suggest that the gap in the means of LAZ is primarily due to the coefficient or effect of the determinants. The variable by variable decomposition of the LAZ gap identified maternal height (coefficient: -3.04 ; 95% CI: 0.35, -6.44), birth weight (coefficient: -0.21; 95% CI: 0.88, -1.30), people per room (coefficient: 0.31; 95% CI: 0.92, -0.30) and energy intake (coefficient: -0.12; 95% CI: 0.22, -0.46) as the top most factors responsible for the mean length-for-age z score difference between stunted and non-stunted at 24 months of age.

DISCUSSION

At month 24 the cohort exhibited 47.9% prevalence of stunting. The figure is similar to Bangladesh Urban Health Survey which reported a 50% prevalence of stunting in under 5 children living in slum areas of Dhaka, Bangladesh.[8]

Our analysis found maternal height to be inversely associated with stunting and exerting the highest relative contribution to LAZ score difference between stunted and non-stunted children at 24 months of age. Comparable findings were reported from studies conducted in similar setting. Results from 109

For peer review only

For peer review only - http://bmjopen.bmj.com/site/about/guidelines.xhtml

Demographic and Health Surveys conducted in 54 LMICs between 1991 and 2008 found maternal stature to be inversely associated with stunting during infancy and childhood.[31] It has also been published that a child, born to a shorter mother, had greater chances of being in a lower length-for-age category.[32] This could be explained by the biological plausibility that shorter mothers may have a poorer health and nutritional status and failed to deliver adequate nutrition to the fetus during pregnancy.[33] This finding signifies the intergenerational effect of malnutrition.

The negative association between birth weight and stunting identifies it as another important correlate of stunting. Moreover, the relative contribution of birth weight on mean length-for-age z score difference between stunted and non-stunted children at their 24 months of age indicates that its contribution did not decline with age. After birth, the mean length-for-age z score experiences a sharp fall, the magnitude of which can be affected by the presence of low birth weight- an indicator for underlying growth hindering factors.[34 35] A meta-analysis of 19 studies showed that under 5 children with low birth weight had 3-fold higher odds of becoming stunted.[36]

Our findings suggest that children living in crowded households were more likely to be stunted. It also contributed to the mean LAZ sore difference between stunted and non-stunted children. This finding corresponds with the elucidations from a study done in Brazil which reported household people per room (OR 2.40; 95% CI: 1.36–4.22) to be associated with a high prevalence of stunting.[37] Another study conducted in India showed that children living in crowded households (family with ≥3 persons per room) had a higher odds of being stunted than the children living in families with 3 members or less.[38] Having a higher number of family members may raise implications on household food insecurity or unavailability of complementary foods. Hence, the relationship warrants a further exploration keeping household food insecurity status as a covariate.

For peer review only

For peer review only - http://bmjopen.bmj.com/site/about/guidelines.xhtml

Though energy intake showed a marginal association with stunting in longitudinal data analysis, it did contribute to the LAZ gap at 24 months. This finding is supported by the multicountry analysis of MAL-ED birth cohort data which reported that lower percent of energy from dietary protein was strongly associated with a decline in length-for-age.[32] Another study done in Zambia revealed that the stunted infants and toddlers had a tendency to take lower energy compared to the non-stunted children.[39]

We found that female children had lower odds of becoming stunted than their male counterparts. A meta-analysis of 16 demographic and health surveys done in sub-Saharan Africa reported boys to be more stunted than girls.[40] Similar results were found from East African herders where parental investments were biased for their female children.[41] On the contrary, studies also reported that in sub-Saharan areas daughters faced a gender biased dietary discrimination that was in favor of boys.[42] A cross-sectional analysis done in Bangladesh, Nepal and India documented that parental preference towards son might affect child undernutrition in this region. [43] However, there is a paucity of data that could elaborate this relation and the variable showed an insubstantial contribution in decomposition analysis.

In longitudinal analysis, having a separate room for kitchen was found to be negatively associated with stunting. Traditional cooking stoves inefficiently combust solid fuels and produce large volumes of indoor smoke.[44] Thus using traditional cooking materials inside the living room acts as a potent source of indoor air pollution by producing life threatening air pollutants.[45] This might have some biologically plausible relation with our finding. Multiple studies have reported air pollution to be associated with low birth weight.[44 46] However, no such report was found regarding stunting and the variable showed near zero contribution in decomposition analysis.

In addition to identifying the determinants of stunting, our study provided additional unique findings that the coefficients or effects of those determinants were primarily responsible for the stunted vs. non

For peer review only - http://bmjopen.bmj.com/site/about/guidelines.xhtml

stunted LAZ gap at 24 months of age. That is the strength of this analysis. But, this study also has its own limitations to report. The study was conducted in a slum area. Hence, the findings of this study may not be applicable to the non-slum setting.

CONCLUSION

The overall goal of the study was to measure the relative contributions of the most predictive correlates of stunting to mean LAZ score difference between stunted and non-stunted children at their 24 months of age. We aimed to generate concrete evidence in helping the policy makers to direct their programs on specific areas of life cycle and to minimize the burden of linear growth faltering. In summary, we have documented that the affect of maternal height and birth weight on linear growth did not decline with age. Our findings indicated the intergenerational transmission of mother’s own nutritional status to her offspring. The significance of this association at the policy level would thus ideally aim for the improvement in the nutritional status of a women during her adolescence and pregnancy, which would translate into positive inputs on the birth weight of her offspring and ultimately, on the linear growth of the child.

Acknowledgement: The authors would like to thank all the participants and their parents for sharing their time and providing consent, information and biological samples necessary for the successful completion of the study.

Contributions: SD, SEA and TA conceived the study and procured funds. MM developed the data collections tools. MAA managed the data set and provided technical support. SD analyzed the data, developed the tables/graphs and wrote the initial draft of the manuscript. MAA, MM, SEA and TA critically reviewed the manuscript and gave intellectual inputs. All authors contributed to the final version of the paper.

For peer review only

For peer review only - http://bmjopen.bmj.com/site/about/guidelines.xhtml

Funding: This paper is made possible by the generous support of the American people through the United States Agency for International Development (USAID) under cooperative agreement no. AID-388-A-17-00006. The views expressed herein are responsibility of the RDM Activity and do not necessarily reflect the views of the U.S. Government or USAID or icddr,b, the implementing agency. The MAL-ED (Etiology, Risk Factors, and Interactions of Enteric Infections and Malnutrition and the Consequences for Child Health) birth cohort study was funded by University of Virginia (UVA) with support from MAL-ED Network Investigators in the Foundation of National Institute of Health (FNIH), Fogarty International Centre (FIC) with overall support from the Bill & Melinda Gates Foundation (BMGF). They also acknowledge the contribution of icddr,b's core donors including Government of the People's Republic of Bangladesh, Global Affairs Canada (GAC), Canada; Swedish International Development Cooperation Agency (SIDA) and the Department for International Development, UKAid for their continuous support and commitment to the icddr,b's research efforts.

Competing interests: The authors do not have any competing interests to declare.

Patient consent: Informed written consent was obtained from the mother or primary caregiver.

Data sharing statement: The data sets generated and/or analyzed during the current study is not made publicly available. However, data inquires or further suggestions for analyses can be made to the corresponding author.

REFERENCES

1. Pradhan M, Sahn DE, Younger SD. Decomposing world health inequality. *Journal of health economics* 2003;22(2):271-93.
2. Black RE, Allen LH, Bhutta ZA, et al. Maternal and child undernutrition: global and regional exposures and health consequences. *The lancet* 2008;371(9608):243-60.
3. Victora CG, Adair L, Fall C, et al. Maternal and child undernutrition: consequences for adult health and human capital. *The lancet* 2008;371(9609):340-57.
4. Grantham-McGregor S, Cheung YB, Cueto S, et al. Developmental potential in the first 5 years for children in developing countries. *The lancet* 2007;369(9555):60-70.

For peer review only - http://bmjopen.bmj.com/site/about/guidelines.xhtml

For peer review only

5. Fanzo J, Hawkes C, Udomkesmalee E, et al. 2018 Global Nutrition Report: Shining a light to spur action on nutrition. 2018
6. Adekanmbi VT, Uthman OA, Mudasiru OM. Exploring variations in childhood stunting in Nigeria using league table, control chart and spatial analysis. *BMC Public Health* 2013;13(1):361.
7. NIPORT M. Bangladesh Demographic and Health Survey BDHS 2014: Key Indicators. *Dhaka, Bangladesh and Calverton, Maryland, USA: National Institute of Population Research and Training (NIPORT), Mitra and Associates, and ICF International* 2014
8. National Institute of Population Research and Training (NIPORT) ICfDDR, Bangladesh (icddr,b), MEASURE Evaluation. Bangladesh Urban Health Survey 2013 Final Report.
9. Waterlow J. Introduction. Causes and mechanisms of linear growth retardation (stunting). *Eur J Clin Nutr* 1994;48(Suppl 1):S1-S4.
10. Hoddinott J, Alderman H, Behrman JR, et al. The economic rationale for investing in stunting reduction. *Maternal & Child Nutrition* 2013;9(S2):69-82.
11. Stewart CP, Iannotti L, Dewey KG, et al. Contextualising complementary feeding in a broader framework for stunting prevention. *Maternal & child nutrition* 2013;9:27-45.
12. Hoddinott J, Alderman H, Behrman JR, et al. The economic rationale for investing in stunting reduction. *Maternal & Child Nutrition* 2013;9:69-82.
13. Ahmed T, Mahfuz M, Islam MM, et al. The MAL-ED cohort study in Mirpur, Bangladesh. *Clinical Infectious Diseases* 2014;59(suppl_4):S280-S86.
14. Lohman TG, Roche AF, Martorell R. Anthropometric standardization reference manual: Human kinetics books Champaign 1988.
15. WHO M. Growth, Reference, Study, Group. *WHO Child Growth Standards: Length/height-for-age, weight-for-age, weight-for-length, weight-for-height and body mass index-for-age: Methods and development Geneva: World Health Organization* 2006
16. Caulfield LE, Bose A, Chandyo RK, et al. Infant feeding practices, dietary adequacy, and micronutrient status measures in the MAL-ED study. *Clinical Infectious Diseases* 2014;59(suppl_4):S248-S54.
17. Islam MM, Sanin KI, Mahfuz M, et al. Risk factors of stunting among children living in an urban slum of Bangladesh: findings of a prospective cohort study. *BMC public health* 2018;18(1):197.
18. Daelmans B, Dewey K, Arimond M. New and updated indicators for assessing infant and young child feeding. *Food and nutrition bulletin* 2009;30(2_suppl2):S256-S62.
19. Psaki SR, Seidman JC, Miller M, et al. Measuring socioeconomic status in multicountry studies: results from the eight-country MAL-ED study. *Population health metrics* 2014;12(1):8.
20. Organization WH. Iron deficiency anaemia: assessment, prevention and control: a guide for programme managers. 2001
21. Grant FK, Suchdev PS, Flores-Ayala R, et al. Correcting for Inflammation Changes Estimates of Iron Deficiency among Rural Kenyan Preschool Children–. *The Journal of nutrition* 2011;142(1):105-11.
22. Richard SA, Barrett LJ, Guerrant RL, et al. Disease surveillance methods used in the 8-site MAL-ED cohort study. *Clinical Infectious Diseases* 2014;59(suppl_4):S220-S24.
23. Baqui AH, Black RE, Yunus M, et al. Methodological issues in diarrhoeal diseases epidemiology: definition of diarrhoeal episodes. *International journal of epidemiology* 1991;20(4):1057-63.
24. Morris SS, Cousens SN, Lanata CF, et al. Diarrhoea—defining the episode. *International Journal of Epidemiology* 1994;23(3):617-23.
25. Lanata CF, Rudan I, Boschi-Pinto C, et al. Methodological and quality issues in epidemiological studies of acute lower respiratory infections in children in developing countries. *International journal of epidemiology* 2004;33(6):1362-72.

For peer review only

For peer review only - http://bmjopen.bmj.com/site/about/guidelines.xhtml

26. Pio A. Standard case management of pneumonia in children in developing countries: the cornerstone of the acute respiratory infection programme. *Bulletin of the World Health Organization* 2003;81(4):298-300.
27. Diggle P, Liang K, Zeger S. Analysis of Longitudinal Data. Clarendon, Press: Oxford, 1994.
28. Jann B. The Blinder-Oaxaca decomposition for linear regression models. *The Stata Journal* 2008;8(4):453-79.
29. Carey VJ, Lumley T, Ripley B. gee: Generalized Estimation Equation solver, R package version 4.13-18, 2015.
30. Hlavac M. oaxaca: Blinder-Oaxaca Decomposition in R. 2014
31. Ízaltin E, Hill K, Subramanian S. Association of maternal stature with offspring mortality, underweight, and stunting in low-to middle-income countries. *Jama* 2010;303(15):1507-16.
32. Investigators M-EN. Childhood stunting in relation to the pre-and postnatal environment during the first 2 years of life: The MAL-ED longitudinal birth cohort study. *PLoS medicine* 2017;14(10):e1002408.
33. Hart N. Famine, maternal nutrition and infant mortality: a re-examination of the Dutch hunger winter. *Population Studies* 1993;47(1):27-46.
34. Shrimpton R, Victora CG, de Onis M, et al. Worldwide timing of growth faltering: implications for nutritional interventions. *Pediatrics* 2001;107(5):e75-e75.
35. Mukhopadhyay K, Mahajan R, Louis D, et al. Longitudinal growth of very low birth weight neonates during first year of life and risk factors for malnutrition in a developing country. *Acta Paediatrica* 2013;102(3):278-81.
36. Christian P, Lee SE, Donahue Angel M, et al. Risk of childhood undernutrition related to small-for-gestational age and preterm birth in low-and middle-income countries. *International journal of epidemiology* 2013;42(5):1340-55.
37. Assis AMdO, Prado MdS, Barreto ML, et al. Childhood stunting in Northeast Brazil: the role of Schistosoma mansoni infection and inadequate dietary intake. *European Journal of Clinical Nutrition* 2004;58(7):1022.
38. Mishra V, Retherford RD. Does biofuel smoke contribute to anaemia and stunting in early childhood? *International Journal of Epidemiology* 2006;36(1):117-29.
39. Hautvast J, Van der Heijden L, Luneta A, et al. Food consumption of young stunted and non-stunted children in rural Zambia. *European journal of clinical nutrition* 1999;53(1):50.
40. Wamani H, Åstrøm AN, Peterson S, et al. Boys are more stunted than girls in sub-Saharan Africa: a meta-analysis of 16 demographic and health surveys. *BMC pediatrics* 2007;7(1):17.
41. Cronk L. Low Socioeconomic Status and Female-Biased Parental Investment: The Mukogodo Example. *American Anthropologist* 1989;91(2):414-29.
42. Leslie J, Essama SB, Ciemins E. Female nutritional status across the life-span in sub-Saharan Africa. 2. Causes and consequences. *Food and Nutrition Bulletin* 1997;18(1):1-12.
43. Raj A, McDougal LP, Silverman JG. Gendered effects of siblings on child malnutrition in South Asia: cross-sectional analysis of demographic and health surveys from Bangladesh, India, and Nepal. *Maternal and child health journal* 2015;19(1):217-26.
44. Haider MR, Rahman MM, Islam F, et al. Association of low birthweight and indoor air pollution: Biomass fuel use in Bangladesh. *Journal of Health and Pollution* 2016;6(11):18-25.
45. Oliveira BFAd, Ignotti E, Hacon SS. A systematic review of the physical and chemical characteristics of pollutants from biomass burning and combustion of fossil fuels and health effects in Brazil. *Cadernos de saude publica* 2011;27(9):1678-98.
46. Chen L, Yang W, Jennison BL, et al. Air pollution and birth weight in northern Nevada, 1991-1999. *Inhalation toxicology* 2002;14(2):141-57.

For peer review only - http://bmjopen.bmj.com/site/about/guidelines.xhtml

429 # TABLES

430 **Table 1:** Maternal-, child-, household-level characteristics and morbidity status of the cohort

n=211 (at 24 month)		All (n=211)	Stunted (n=101)	Not stunted (n=110)	
		n (%)			p-value
Maternal-, child- and household-level characteristics					
Sex	Male	108 (51.2)	57 (56.4)	51 (46.4)	0.1
Exclusive breast feeding*	Yes	23 (10.9)	12 (11.9)	11 (10.0)	0.66
Household head's sex	Male	195 (92.4)	95 (94.1)	100 (90.9)	0.83
Mother's age at birth (years)	<18y	3 (1.4)	0 (0.0)	3 (2.7)	0.2
	18-30y	178 (84.4)	84 (83.2)	94 (85.5)	
	>30y	30 (14.2)	17 (16.8)	13 (11.8)	
Mother's age at first pregnancy	<18y	84 (39.8)	34 (33.7)	50 (45.5)	0.08
Maternal education	No education	41 (19.4)	22 (21.8)	19 (17.3)	0.71
	<5y	93 (44.1)	43 (42.6)	50 (45.5)	
	>5y	77 (36.5)	36 (35.6)	41 (37.3)	
Separate room for a kitchen	Yes	17 (8.1)	4 (4.0)	13 (11.8)	0.04
Monthly family income	<100 USD	94 (44.5)	52 (51.5)	42 (38.2)	0.05
Households have chicken or ducks	Yes	13 (6.2)	5 (5.0)	8 (7.3)	0.48

For peer review only - http://bmjopen.bmj.com/site/about/guidelines.xhtml

Treat water to make it safe	Yes	135 (64.0)	56 (55.4)	78 (70.9)	0.02
		Mean (SD)			
Exclusive breast feeding days (ebf days)		102.00 (57.7)	105.00 (56.9)	99.6 (58.5)	<0.00
WAMI score**		0.53 (0.13)	0.50 (0.13)	0.55 (0.13)	0.17
People per room		3.57 (1.18)	3.83 (1.32)	3.33 (0.98)	<0.00
Birth weight (kg)		2.82 (0.42)	2.70(0.39)	2.92(0.42)	<0.00
Maternal height (cm)		149.03 (5.05)	147.34 (4.85)	150.54 (4.76)	<0.00
Morbidity episodes/child-year					
Diarrhea		3.63	4.03	3.43	0.08
ALRI		0.47	0.54	0.46	0.98
Fever		8.06	8.78	7.87	0.74
Cough		9.69	9.64	9.83	0.02

* Excluding breast feeding: Exclusive breastfeeding is defined as no other food or drink, not even water, except breast milk (including milk expressed or from a wet nurse) for 6 months of life, but allows the infant to receive ORS, drops and syrups (vitamins, minerals and medicines)

**The WAMI score (ranging from 0 to 1) is a measure of household socioeconomic status including access to improved Water, sanitation and hygiene (WASH), Assets, Maternal education, and Income.

Table 2: Determinants of stunting among children: results of Generalized Estimating Equation modeling

(*Dependent variable: Stunted no vs.yes)*

	Unadjusted OR (95% CI	p-value	Adjusted OR (95% CI	p-value
**Energy intake*(kcal)**	**1.001 (1.001 -1.001)**	**0.01**	**1.001 (1.001-1.002)**	**<0.00**

For peer review only - http://bmjopen.bmj.com/site/about/guidelines.xhtml

Ebf days	1.002 (1.001 - 1.003)	<0.00	1.002 (1.002-1.002)	0.81
Minimum acceptable diet	0.74 (0.64 - 0.85)	<0.00	0.89 (0.72 - 1.09)	0.25
Cough episodes (per child-year)	0.90 (0.82 - 0.98)	<0.00	0.97 (0.86 - 1.10)	0.67
Anemia**	1.45 (1.26 - 1.69)	<0.00	1.09 (0.90 - 1.32)	0.40
**Gender**	**0.70 (0.61 - 0.80)**	**<0.00**	**0.47 (0.38 - 0.58)**	**<0.00**
**Maternal height (cm)***	**0.89 (0.88 - 0.91)**	**<0.00**	**0.87 (0.86 - 0.89)**	**<0.00**
**Separate room for a kitchen**	**0.37 (0.28 - 0.50)**	**<0.00**	**0.21 (0.12 - 0.38)**	**<0.00**
WAMI	0.06 (0.04 - 0.11)	<0.00	0.39 (0.15 - 1.03)	0.06
**People per room***	**1.34 (1.26 - 1.42)**	**<0.00**	**1.48 (1.35 - 1.62)**	**<0.00**
**Birth weight (kg)***	**0.18 (0.15 - 0.22)**	**<0.00**	**0.11 (0.09 - 0.15)**	**<0.00**
Serum Zinc (mmol/L)	0.94 (0.91 - 0.98)	<0.00	0.99 (0.95 - 1.04)	0.79

OR= Odds ratio; CI= Confidence intervals

*per one unit change

** Anemia is defined as a hemoglobin concentration <110 g/L

Table 3: Relative contribution of the selected variables on mean length-for-age Z score difference between stunted and non-stunted children at 24 month

	Endowment	Coefficient	Interaction

For peer review only - http://bmjopen.bmj.com/site/about/guidelines.xhtml

	Coef.	95% CI		Coef.	95% CI		Coef.	95% CI	
		Upper	Lower		Upper	Lower		Upper	Lower
Overall	0.26	0.41	0.10	1.34	1.50	1.19	-0.12	0.03	-0.26
Energy intake (kcal)	0.00	0.03	-0.03	-0.12	0.22	-0.46	0.00	0.03	-0.03
Gender	0.01	0.02	-0.01	0.02	0.11	-0.07	0.00	0.01	-0.02
Maternal height (cm)	0.10	0.18	0.02	-3.04	0.35	-6.44	-0.07	0.02	-0.15
Separate kitchen	0.00	0.03	-0.03	0.01	0.03	-0.02	0.02	0.06	-0.03
People per room	0.06	0.12	0.00	0.31	0.92	-0.30	-0.04	0.04	-0.12
Birth weight (kg)	0.08	0.17	-0.01	-0.21	0.88	-1.30	-0.02	0.07	-0.11

Figure legends

Figure 1

Title: Patterns of macronutrient intake from 9 to 24 months of age

Legends: Mean macronutrient intake per day (grams), Months of age, Animal protein, Carbohydrate, Fat, Total protein, Stunted, Not stunted

Figure 2

Title: Prevalence of stunting among children who did not take Minimum Acceptable Diet (MAD) and those who took it (MAD: For breastfed children MAD is defined as who had at least the minimum dietary diversity and the minimum meal frequency during the previous day and for non-breastfed children it is defined as who received at least 2 milk feedings and had at least the minimum dietary diversity not including milk feeds and the minimum meal frequency during the previous day)

Legends: Prevalence of stunting (95 % CI), Months of age, Did not take MAD, Took MAD

For peer review only - http://bmjopen.bmj.com/site/about/guidelines.xhtml

456 **Supplementary figure 1**

457 Title: Serum zinc, ferritin and hemoglobin levels of stunted and non-stunted children at 7, 15, and 24
458 months of age

459 Legends: Zinc (mmol/L), Ferritin (ug /L), Hemoglobin (g/dL), Stunted, No, Yes

460 **Supplementary figure 2**

461 Title: Coefficient plot of relative contributions of the selected variables on mean LAZ score difference
462 between stunted and non-stunted children at 24 months of age

463 Legends: Coefficients of relative contribution, Energy intake, Gender, Maternal height, Separate kitchen,
464 Crowding, Birth weight

465

For peer review only

For peer review only - http://bmjopen.bmj.com/site/about/guidelines.xhtml

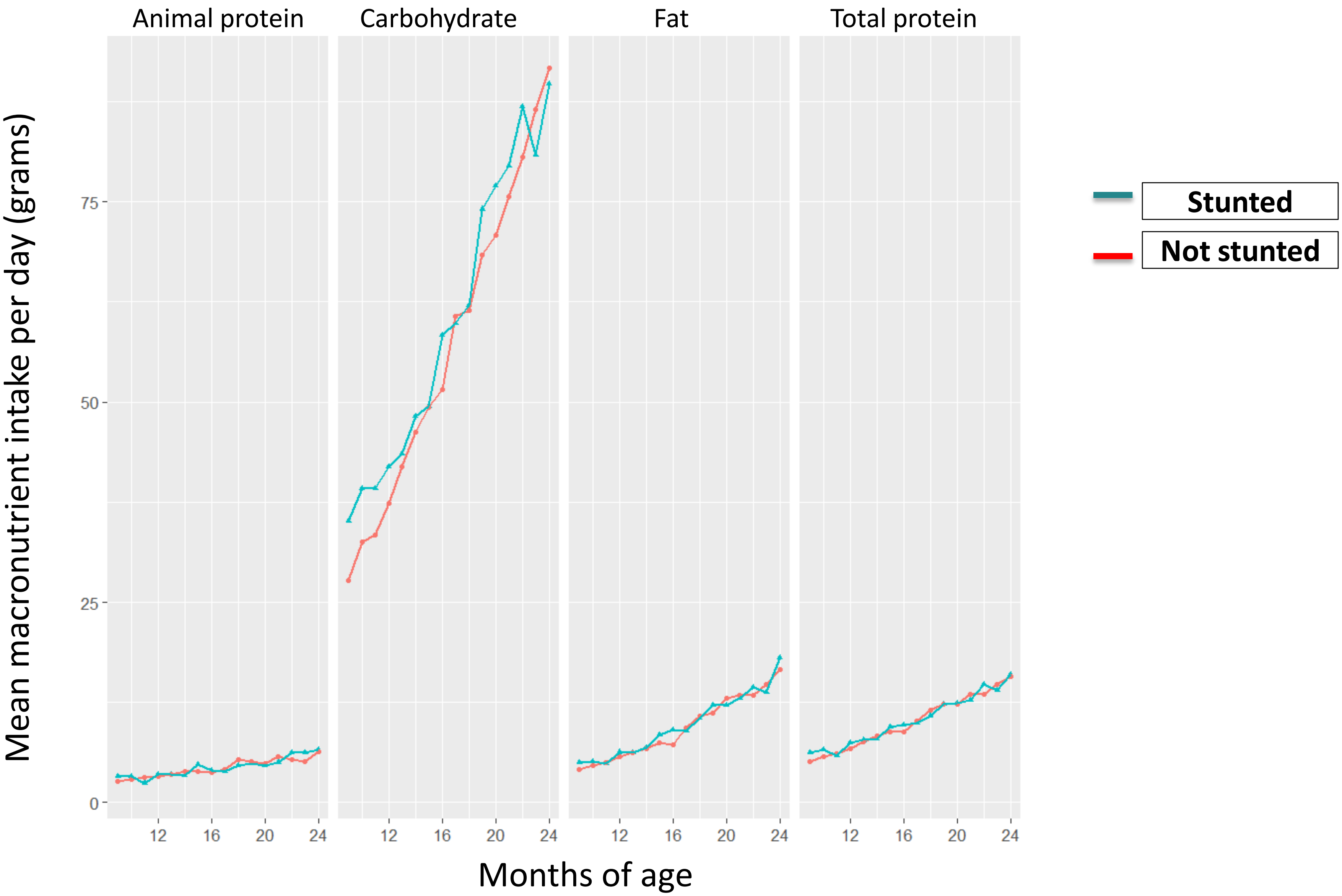
Animal protein
Carbohydrate
Fat
Total protein
Mean macronutrient intake per day (grams)
75
50
25
0
12
16
20
24
Months of age
Stunted
Not stunted

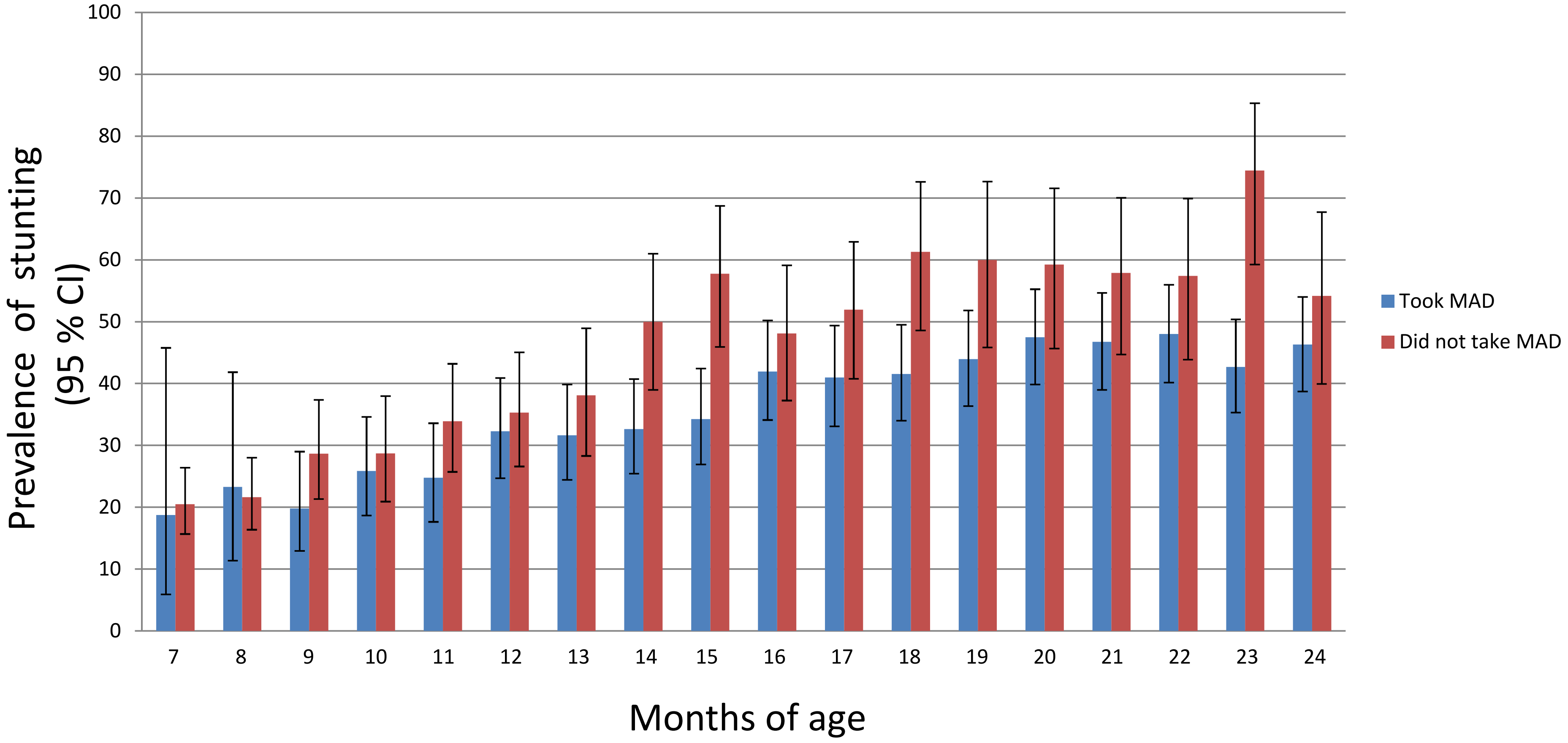
Prevalence of stunting (95 % CI)
100
90
80
70
60
50
40
30
20
10
0
7
8
9
10
11
12
13
14
15
16
17
18
19
20
21
22
23
24
Months of age
Took MAD
Did not take MAD

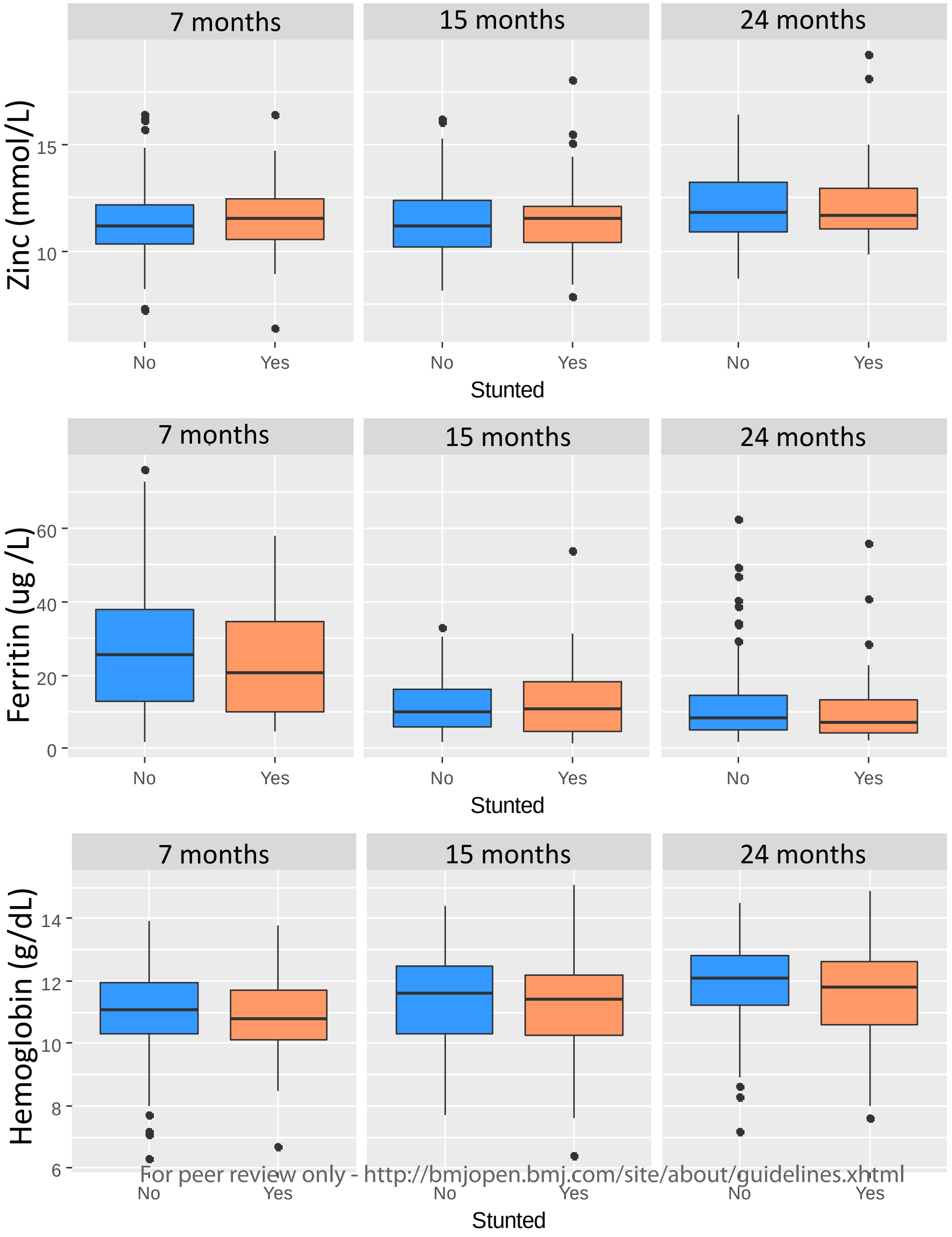
7 months
15 months
24 months
Zinc (mmol/L)
15
10
No
Yes
Stunted
Ferritin (ug /L)
60
40
20
0
Hemoglobin (g/dL)
14
12
10
8
6

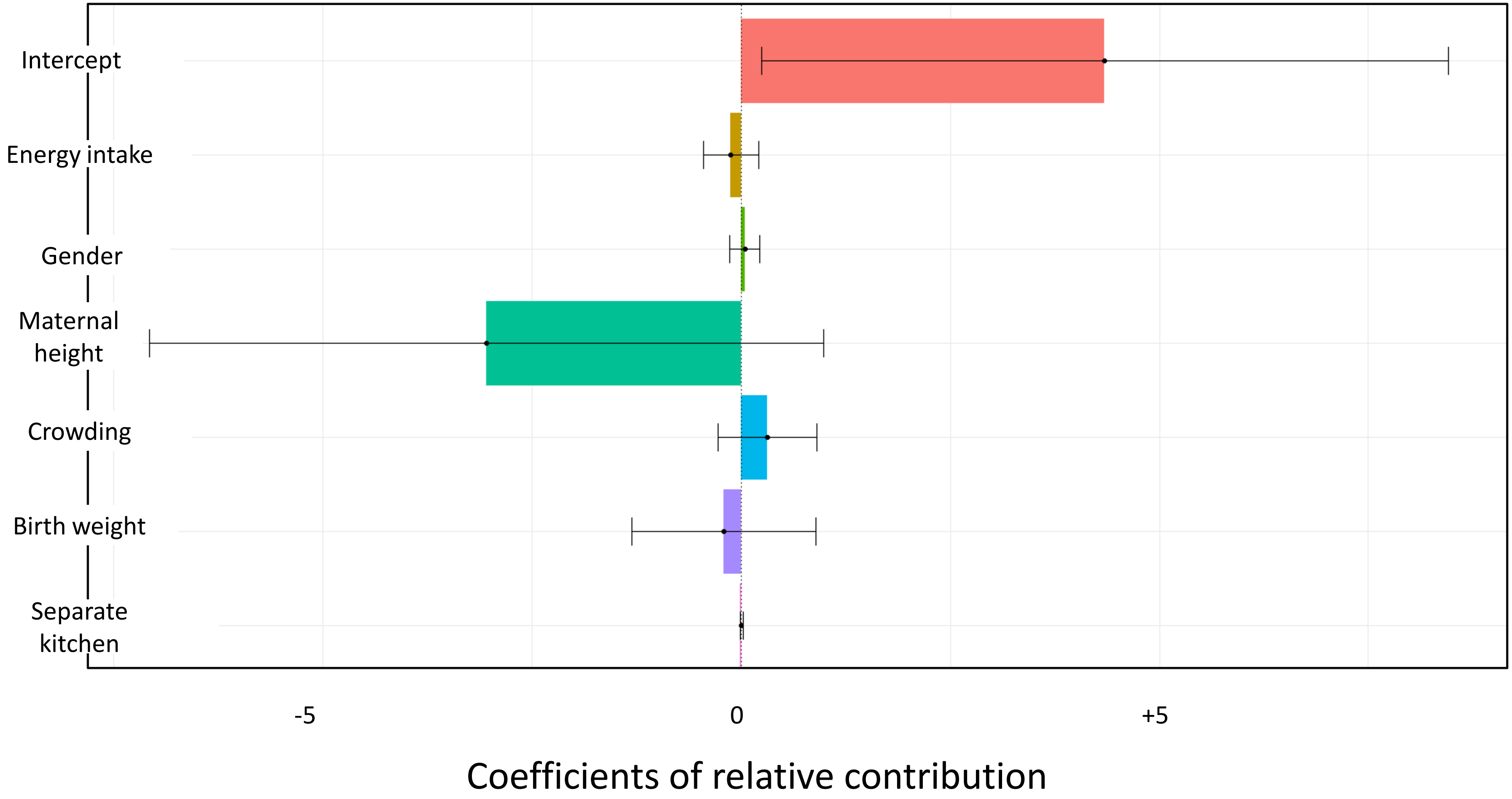
Intercept
Energy intake
Gender
Maternal height
Crowding
Birth weight
Separate kitchen
-5
0
+5
Coefficients of relative contribution

STROBE (Strengthening The Reporting of OBservational Studies in Epidemiology) Checklist

A checklist of items that should be included in reports of observational studies. You must report the page number in your manuscript where you consider each of the items listed in this checklist. If you have not included this information, either revise your manuscript accordingly before submitting or note N/A.

Note: An Explanation and Elaboration article discusses each checklist item and gives methodological background and published examples of transparent reporting. The STROBE checklist is best used in conjunction with this article (freely available on the Web sites of PLoS Medicine at http://www.plosmedicine.org/, Annals of Internal Medicine at http://www.annals.org/, and Epidemiology at http://www.epidem.com/). Information on the STROBE Initiative is available at www.strobe-statement.org.

Section and Item	Item No.	Recommendation	Reported on Page No.
**Title and Abstract**	1	(*a*) Indicate the study's design with a commonly used term in the title or the abstract	1,2
		(*b*) Provide in the abstract an informative and balanced summary of what was done and what was found	1,2
**Introduction**			
Background/Rationale	2	Explain the scientific background and rationale for the investigation being reported	3-4
Objectives	3	State specific objectives, including any prespecified hypotheses	4
**Methods**			
Study Design	4	Present key elements of study design early in the paper	4
Setting	5	Describe the setting, locations, and relevant dates, including periods of recruitment, exposure, follow-up, and data collection	4
Participants	6	(*a*) *Cohort study*—Give the eligibility criteria, and the sources and methods of selection of participants. Describe methods of follow-up	4
		Case-control study—Give the eligibility criteria, and the sources and methods of case ascertainment and control selection. Give the rationale for the choice of cases and controls	n/a
		Cross-sectional study—Give the eligibility criteria, and the sources and methods of selection of participants	n/a
		(*b*) *Cohort study*—For matched studies, give matching criteria and number of exposed and unexposed	n/a
		Case-control study—For matched studies, give matching criteria and the number of controls per case	n/a
Variables	7	Clearly define all outcomes, exposures, predictors, potential confounders, and effect modifiers. Give diagnostic criteria, if applicable	5-6

For peer review only

Section and Item	Item No.	Recommendation	Reported on Page No.
Data Sources/ Measurement	8*	For each variable of interest, give sources of data and details of methods of assessment (measurement). Describe comparability of assessment methods if there is more than one group	5-6
Bias	9	Describe any efforts to address potential sources of bias	7
Study Size	10	Explain how the study size was arrived at	4
Quantitative Variables	11	Explain how quantitative variables were handled in the analyses. If applicable, describe which groupings were chosen and why	7
Statistical Methods	12	(*a*) Describe all statistical methods, including those used to control for confounding	7
		(*b*) Describe any methods used to examine subgroups and interactions	n/a
		(*c*) Explain how missing data were addressed	7
		(*d*) *Cohort study*—If applicable, explain how loss to follow-up was addressed	7
		Case-control study—If applicable, explain how matching of cases and controls was addressed	n/a
		Cross-sectional study—If applicable, describe analytical methods taking account of sampling strategy	n/a
		(*e*) Describe any sensitivity analyses	n/a
**Results**			
Participants	13*	(a) Report numbers of individuals at each stage of study—eg numbers potentially eligible, examined for eligibility, confirmed eligible, included in the study, completing follow-up, and analysed	7
		(b) Give reasons for non-participation at each stage	n/a
		(c) Consider use of a flow diagram	n/a
Descriptive Data	14*	(a) Give characteristics of study participants (eg demographic, clinical, social) and information on exposures and potential confounders	7,8
		(b) Indicate number of participants with missing data for each variable of interest	n/a
		(c) *Cohort study*—Summarise follow-up time (eg, average and total amount)	4
Outcome Data	15*	*Cohort study*—Report numbers of outcome events or summary measures over time	7
		Case-control study—Report numbers in each exposure category, or summary measures of exposure	
		Cross-sectional study—Report numbers of outcome events or summary measures	

For peer review only

Section and Item	Item No.	Recommendation	Reported on Page No.
Main Results	16	(*a*) Give unadjusted estimates and, if applicable, confounder-adjusted estimates and their precision (eg, 95% confidence interval). Make clear which confounders were adjusted for and why they were included	9,17
		(*b*) Report category boundaries when continuous variables were categorized	n/a
		(*c*) If relevant, consider translating estimates of relative risk into absolute risk for a meaningful time period	n/a
Other Analyses	17	Report other analyses done—eg analyses of subgroups and interactions, and sensitivity analyses	n/a
**Discussion**			
Key Results	18	Summarise key results with reference to study objectives	10,11
Limitations	19	Discuss limitations of the study, taking into account sources of potential bias or imprecision. Discuss both direction and magnitude of any potential bias	11
Interpretation	20	Give a cautious overall interpretation of results considering objectives, limitations, multiplicity of analyses, results from similar studies, and other relevant evidence	10,11
Generalisability	21	Discuss the generalisability (external validity) of the study results	10,11
**Other Information**			
Funding	22	Give the source of funding and the role of the funders for the present study and, if applicable, for the original study on which the present article is based	12

*Give information separately for cases and controls in case-control studies and, if applicable, for exposed and unexposed groups in cohort and cross-sectional studies.

Once you have completed this checklist, please save a copy and upload it as part of your submission. DO NOT include this checklist as part of the main manuscript document. It must be uploaded as a separate file.

For peer review only
